# An mRNA vaccine encoding the Ebola virus glycoprotein induces high neutralizing antibody titers and provides strong protection against lethal infections in mouse models

**DOI:** 10.3389/fimmu.2025.1682418

**Published:** 2026-01-05

**Authors:** Zachary R. Stromberg, Elvia E. Silva, Sara C. Johnston, Dylan M. Johnson, Dominique Hall, Emmanuel F. Adjei, John A. Altin, Abisola Abisoye-Ogunniyan, Travis Gollott, Wei He, Iris K. A. Jones, Steve Kwilas, Sean J. Lund, Heather L. Mead, Georgia A. Nelson, Lisa Ouyang, Sandra K. G. Peters, Jennifer L. Schwedler, Caleb Z. Trecazzi, Matthew W. Turner, Chunyan Ye, Steven B. Bradfute, Nicholas O. Fischer, Jay W. Hooper, Jason T. Ladner, Amy Rasley, Oscar A. Negrete

**Affiliations:** 1 Chemical and Biological Signatures Group, Pacific Northwest National Laboratory, Richland, WA, United States; 2 Department of Systems Biology, Sandia National Laboratories, Livermore, CA, United States; 3 Virology Division, United States Army Medical Research Institute of Infectious Diseases, Fort Detrick, MD, United States; 4 Biosciences and Biotechnology Division, Lawrence Livermore National Laboratory, Livermore, CA, United States; 5 The Pathogen and Microbiome Institute, Northern Arizona University, Flagstaff, AZ, United States; 6 Department of Biological Sciences, Northern Arizona University, Flagstaff, AZ, United States; 7 The Translational Genomics Research Institute, Flagstaff, AZ, United States; 8 Department of Internal Medicine, Center for Global Health, University of New Mexico Health Sciences Center, Albuquerque, NM, United States; 9 Department of Molecular and Cell Biology, University of California, Merced, CA, United States

**Keywords:** Ebola virus, mRNA vaccines, lipid nanoparticles, VSV-vectored vaccines, DNA vaccines, needle-free injection, PepSeq

## Abstract

Ebola virus (EBOV) is the causative agent of Ebola disease (EBOD), a viral hemorrhagic fever with a notably high case fatality rate. Current treatments for EBOD are limited to monoclonal antibodies or two licensed viral vector vaccines, a recombinant vesicular stomatitis virus (rVSV)-vectored vaccine or an adenovirus and modified vaccinia Ankara regimen. However, comparisons of protection, efficacy, and durability with alternative nucleotide platforms remain understudied. Here, we evaluated the immunogenicity of an mRNA vaccine expressing the EBOV glycoprotein (GP) in parallel with rVSV- and DNA-based vaccine platforms. The mRNA EBOV-GP vaccine, formulated in lipid nanoparticles, elicited significantly higher levels of total IgG and neutralizing antibody titers compared to the rVSV-EBOV-GP vaccine. Linear antibody epitope analysis indicated a preference for targeting the mucin-like domain in EBOV-GP1 following rVSV-based vaccination, while the mRNA platform distinctly targeted the internal fusion loop of EBOV-GP2. After characterizing the immunogenicity of the mRNA vaccine, two models of EBOD were used to demonstrate its protective efficacy: a surrogate rVSV-based challenge model of EBOD using type-I interferon deficient C57BL/6 mice and infection of BALB/c mice with authentic mouse-adapted EBOV. In both studies, the EBOV mRNA vaccine fully protected the mouse cohorts against morbidity and mortality. Additionally, the EBOV mRNA vaccine produced greater neutralizing antibody titers compared to the DNA EBOV-GP vaccine. These results suggest that an mRNA vaccine expressing EBOV-GP can induce robust, functional humoral responses that are protective against EBOD, warranting further development as an alternative to, or as part of a vaccine strategy including, viral vectored vaccines.

## 1 Introduction

Ebola disease (EBOD) is a reemerging viral hemorrhagic fever (VHF) that has significantly affected global health due to recurrent outbreaks (1, 2). EBOD is caused by infection with several highly pathogenic members of the *Orthoebolavirus* genus: *Orthoebolavirus zairense* (Ebola virus [EBOV]), *Orthoebolavirus sudanense* (Sudan virus [SUDV]), and *Orthoebolavirus bundibugyoense* (Bundibugyo virus [BDBV]). Outbreaks of EBOV and SUDV result in severe disease, with case-fatality rates at 67% and 48%, respectively (3, 4). The clinical presentation of EBOD can include high fever, bleeding, coagulopathy, headaches, neurological presentation, gastrointestinal distress, fluid loss, and shock leading to systemic inflammation, cytokine storm, multiorgan failure, and high mortality rate (3–8). EBOD is transmittable through direct contact and appears to also spread through droplet infection via mucus membranes; high nosocomial infection rates may be explained by aerosol generating procedures and fomites may also play a role (9–11).

There are currently two vaccines licensed for the prevention of EBOD caused by EBOV (EVD). The first is based on a recombinant vesicular stomatitis virus (rVSV) where the EBOV glycoprotein (GP) gene has replaced the VSV glycoprotein gene, resulting in the rVSVΔG-ZEBOV-GP construct distributed under the trade name ERVEBO^®^ (Merck, rVSVΔG−ZEBOV−GP; licensed in US/EU). Additionally, a two-dose regimen that begins with an adenovirus serotype 26 vectored EBOV-GP (Ad26.ZEBOV), followed by a modified vaccinia Ankara virus vectored vaccine containing EBOV-GP, SUDV-GP, Tai Forest virus (TAFV) nucleoprotein, and Marburg virus (MARV) GP, marketed as MVA-Filo (Zabdeno^®^, the Ad26.ZEBOV component,/Mvabea^®^, the MVA-Filo component; licensed in the EU [Johnson & Johnson]), is also clinically approved (12–14). ERVEBO^®^ is based on a replication competent live attenuated vaccine (LAV) that utilizes the VSV replicative machinery combined with EBOV-GP to mediate entry into host cells and fusion with endosomal membranes for entry into the cytosolic compartment (15, 16). Ad26.ZEBOV and MVA-Filo each are single-cycle infectious particles that are non-replicative (17, 18). ERVEBO^®^ is highly effective in an outbreak response setting as a ring vaccination strategy where concentric circles of the contact tracing from an exposed person are vaccinated to prevent disease transmission (19, 20). Alternatively, Zabdeno/Mvabea^®^ can be used as prophylactic vaccine as the two doses are spaced 3 months apart, and given its multivalency, may offer broader potential cross-coverage. However, despite the inclusion of additional Filovirus GP antigens, Zabdeno/Mvabea^®^ is only licensed for the prevention of EVD caused by EBOV.

While both licensed vaccines have significant advantages, vector-based immunity is also a concern for both rVSV-EBOV-GP and Ad26.ZEBOV/MVA-Filo (21–24). The role of vector-based immunity in the VSV platform has not been fully elucidated and warrants consideration, particularly as the platform is being considered for other hemorrhagic fever diseases (23, 25–28). Clinical trials have attempted booster doses of rVSV-EBOV-GP administered 56 days after the primary dose, resulting in similar humoral and cellular responses compared to a single dose (29–31). Although VSV- specific humoral and cellular responses have been observed in vaccinees (32, 33), the potential implications of vector-based immunity in limiting booster efficacy have yet to be explored. Considering the waning neutralizing response in rVSV-EBOV-GP vaccinees (33, 34), and the reduced efficacy at 12 months post vaccination in NHP models of infection (35), vector-based immunity, particularly for the use case of booster doses, warrants additional investigation. For Ad26 EBOD vaccines, preliminary evidence from an NHP model suggests that vector-based immunity does not diminish the response to booster doses or subsequent vaccination (36). Additionally, innate immune responses to VSV replication likely play a role in the rapid time-to-protection and may partially explain some of the efficacy of ERVEBO in EBOV infected individuals (37, 38). However, vaccine-mediated antibody titer, and protection in NHP models, wanes relatively quickly and the role of vector immunity in booster doses is not fully understood (35, 39–41). Several comprehensive reviews have noted that correlates of EBOD vaccine efficacy vary by vaccine formulation, route of administration, route of infection, and are likely to remain somewhat cryptic (42–44). While there is a clear link between vaccine derived neutralizing antibody titers and protective efficacy, cellular immunity, including Fc effector function can also play a significant role in successful vaccination (42, 43).

Alternative vaccine platforms that address some limitations of viral vector systems need to be considered as potential preventative vaccines or as booster doses in conjunction with existing vaccines to protect at-risk populations in EBOV-endemic regions. mRNA vaccines were first approved for use in humans during the COVID-19 pandemic which demonstrated the rapid and scalable manufacturing advantages of this platform in addressing emerging viral outbreaks (45–47). Similarly, DNA vaccines offer several advantages, including similar manufacturing convenience to mRNA vaccines while being stable at room temperature, which simplifies storage and distribution. DNA and mRNA vaccines, except for mRNA replicon vaccines, typically provide antigenic proteins such as EBOV-GP without additional accessory proteins. As a result, nucleic acid vaccines can be rapidly designed and produced in response to emerging infectious diseases, incorporating strain specific emerging antigenic changes, making them a valuable tool in public health outbreak response. While nucleic acid vaccines have been investigated for the prevention of EBOD, complete immune response characterization in various animal models remain to be studied.

The use of multiple booster doses of SARS-CoV-2 mRNA vaccines suggest that mRNA platforms may be well suited to this use (48). Importantly, while all these platforms have been tested for protective efficacy, the research designs employed are not optimal to make direct comparisons of LAV, DNA, and mRNA vaccination strategies. Recent reviews have thoroughly explored the use-case scenarios for EBOD vaccination based on different vaccine platforms, and target product profiles have been developed to aid in identifying key vaccine properties that play a role in decision criteria (49–52).

In this study, we experimentally investigated the similarities and differences of immune responses stimulated by mRNA, rVSV, and DNA platform technologies for an EBOV vaccine. Comparisons of immunogenicity and protective efficacy were conducted in mouse models. This work emphasizes the importance of simultaneously comparing vaccine platforms to identify the distinct advantages of each technology. By doing so, we underscore the necessity of further developing the mRNA platform for targeting EBOV.

## 2 Results

### 2.1 Synthesis and expression analysis of EBOV-GP from mRNA, rVSV, and DNA vaccine constructs

An mRNA vaccine encoding codon optimized EBOV-GP from the Mayinga strain with the native signal sequence replaced with the human Igκ signal sequence, and 5’- and 3’- UTRs optimized for expression was synthesized as previously described (**Supplementary Figure S1A**, **Supplementary Table S1**) (53, 54). The EBOV-GP RNA editing site was removed to ensure production of only full-length GP. Denaturing agarose gel electrophoresis confirmed the purified mRNA product was the expected size (**Supplementary Figure S1B**). Following transfection of the mRNA into A549 cells, EBOV-GP was expressed in cells and detected in cell lysates, but not supernatants, indicating the membrane bound form (**Supplementary Figure S1C**). Intracellular immunofluorescent staining with poly-clonal anti-EBOV-GP antibody (**Supplementary Figure S1D**) also confirmed antigen expression by mRNA. The mRNAs were synthesized with a 5’ cap, poly-A tail, and with or without the replacement of uridine with N1-methylpseudouridine (N1MePsU), a modification aimed at reducing innate immune activation and RNA degradation (48, 54, 55). Dynamic light scattering revealed that the LNPs were approximately 100 nm in size and exhibited monodispersity for both encapsulated EBOV-GP mRNA and control firefly luciferase (FLuc) encoding mRNA. The final encapsulation efficiencies were 98.13% for FLuc mRNA and 98.33% for EBOV-GP mRNA (**Supplementary Table S2**). Recombinant VSV-EBOV-GP was generated by reverse genetics in 293T cells, passaged in Vero cells, and purified by ultracentrifugation to generate vaccine aliquots (**Supplementary Figure S2A**). EBOV-GP protein was detected in rVSV-EBOV-GP aliquots by western blot analysis (**Supplementary Figure S2B**). Lastly, flow cytometry was conducted to assess the expression of EBOV-GP following transfection with the DNA vaccine expression plasmid. Twenty-four hours post-transfection, about one-third of 293T cells stained positive for EBOV-GP using polyclonal anti-EBOV rabbit serum (**Supplementary Figure S3**).

### 2.2 EBOV mRNA vaccination generates protective antibody titers but lower T cell responses when compared against rVSV-EBOV-GP

**Figure 1:**
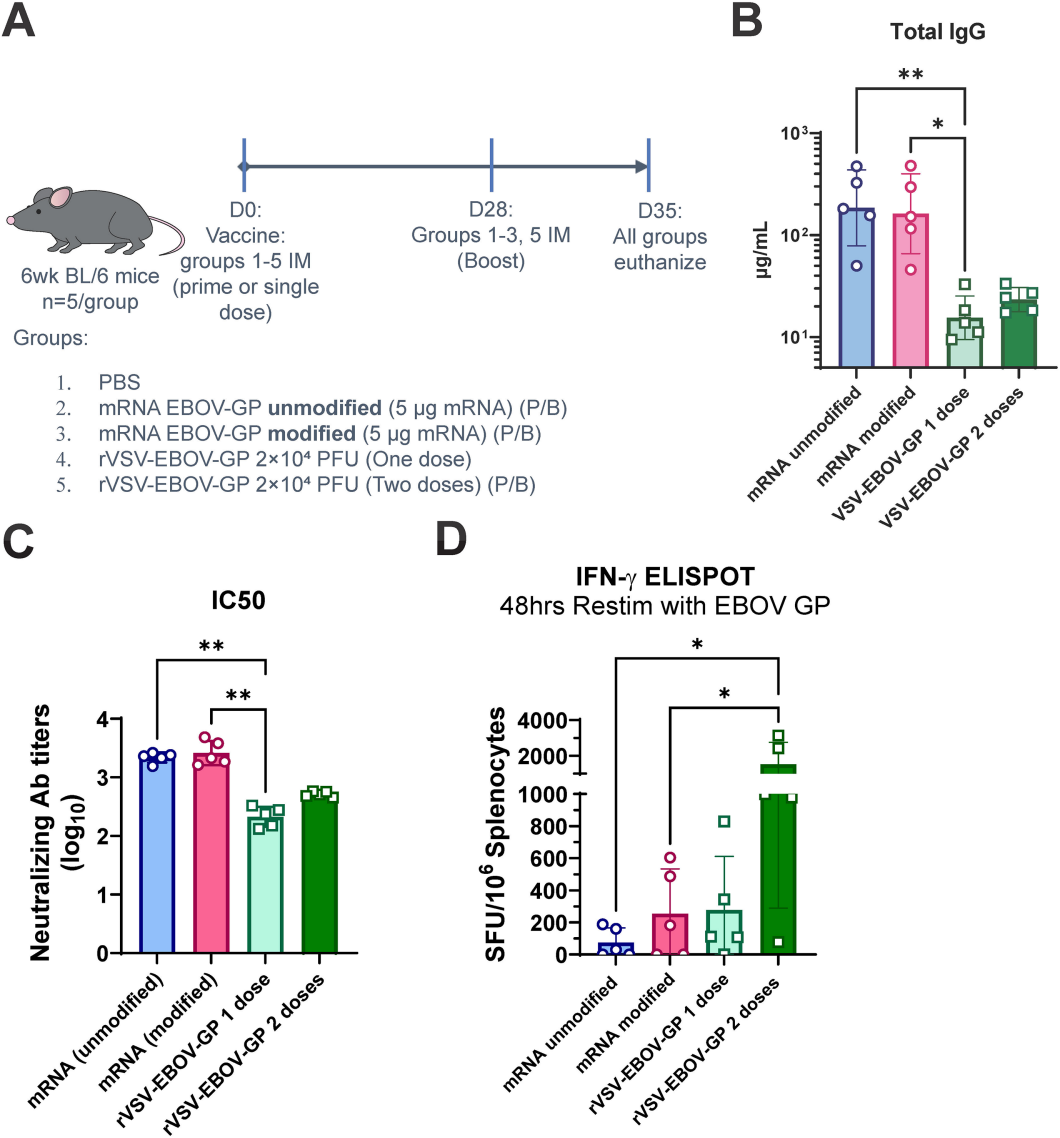
Antibody and T cell responses following EBOV mRNA and rVSV vaccinations. **(A)** In this experimental design, C57BL6/J mice (5/group) were immunized via intramuscular injection with PBS, unmodified or N1MePsU-modified mRNA encoding EBOV-GP formulated in LNPs, or rVSV-EBOV-GP. The rVSV-based vaccine was dosed at 2×10^4^ PFU, whereas mRNA LNPs were dosed at 5 μg per mouse, 2.5 μg per leg. All groups were immunized on day 0 and relevant groups boosted at day 28, except for one condition where rVSV-EBOV-GP was given only as a single dose. On day 35, mice were euthanized and spleens and sera were collected. **(B)** Total anti-EBOV-GP IgG concentration in sera was quantified via ELISA. Concentrations were determined using a standard curve. **(C)** Sera were also analyzed for neutralizing antibody titers. Serially diluted amounts of serum were plated on Vero cells along with a reporter VSV-EBOV-GP expressing GFP. Fluorescence was quantified via Tecan Cyto Spark counting, with % infected determined by green signal/blue nuclear stain signal. Samples plotted as log10 neutralizing antibody titers. **(D)** Splenocytes from one week post boost immunization were incubated with whole EBOV-GP protein for 2 days at 37 °C with 5% CO_2_ and produced IFN-γ signal was quantified by brightfield chromogenic enzymatic ELISpot. SFU=spot forming units. N = 5 per group. *p<0.05, **p<0.01, via Kruskal-Wallis test with Dunns multiple comparison test. Lack of illustrated comparison bar between comparison groups *indicates no significance* between groups, where p>0.05. We omit non-significance comparison labels for clarity of figures.

To allow direct comparisons of immune responses generated by rVSV- and mRNA-based vaccines, C57BL/6 mice were immunized. Although both BALB/c and C57BL/6 mouse strains have been used to evaluate EBOV vaccine candidates (56, 57), the rVSV platform has been extensively characterized in BALB/c mice with a 2.0×10^4^ dose (15, 58–62). To fill these knowledge gaps, C57BL/6 mice were chosen for this initial comparison. Both N1MePsU-modified and unmodified mRNA vaccines, formulated into LNPs, were included. All mice were primed with their respective vaccine formulations at day 0, and all but one group were boosted 28 days later. The remaining group was vaccinated with rVSV-EBOV-GP without a booster. This prime only rVSV group was provided as an ERVEBO-like clinically relevant regimen group. A phosphate buffered saline (PBS) mock vaccinated negative control group was also included. Mice were euthanized 35 days following the primary dose (7 days following the booster) and assayed for humoral and cellular immune responses similar to previous studies from our group (63) (**Figure 1A**). Overall IgG response concentration found in the serum was significantly higher in both the unmodified mRNA and modified mRNA immunized mice compared to the amount induced by a single dose (prime only) of the rVSV-EBOV-GP platform. The unmodified mRNA had a ~10-fold increase while the modified mRNA had a 9.1-fold increase in serum IgG concentration compared to the prime+boost rVSV-EBOV-GP regimen, though both not statistically significant (**Figure 1B**). Next, to determine the neutralizing capacity of serum from vaccinated mice, an rVSV-EBOV-GP expressing GFP was employed. The mRNA vaccines, whether modified or unmodified, had greater neutralizing antibody titers on average compared to the rVSV-EBOV-GP vaccine conditions (**Figure 1C**).

However, as functional T cells are also important for protection against EBOD (57, 64–66), we ascertained the T cell cytokine production against the EBOV-GP antigen via enzyme-linked immunosorbent spot (ELISpot) assay. One week after the boost immunization, spleens were isolated from all groups and cultured as single cell suspensions along with whole protein antigen for 48 h. The ELISpot assay detected high interferon gamma (IFN-γ) positive T cell responses following a two-dose regimen of rVSV-EBOV-GP, moderate responses from a single dose of rVSV-EBOV-GP or with N1MePsU-modified mRNA LNPs, and relatively low responses from unmodified mRNA LNPs (**Figure 1D**, **Supplementary Figure S4**). These experiments illustrate that while rVSV-EBOV-GP leads in production of interferon gamma when provided whole protein for processing, the nucleotide-based mRNA LNP vaccines generated superior serum IgG concentrations and neutralization capacity.

### 2.3 EBOV mRNA vaccination produces a distinct breadth of antibodies against EBOV-GP

PepSeq is a technology that links peptides to DNA barcodes, en masse, to allow epitope-resolved characterization of the antibody repertoire generated in response to vaccination (67, 68). While the quantity of antibodies found in specific tissues including serum can be indicative of the level of humoral protection generated from vaccination or infection, the quality of these antibodies is an important factor in overall protective efficacy of vaccines. Specifically, the capacity of the antibody pool to generate a variety of isotypes and paratopes provides advantages by targeting multiple regions of the antigen (reducing the potential for antibody escape). To further investigate the nature of vaccine induced humoral immunity, serum collected from immunized mice one week after boost, or 35 days total after the prime vaccination, was characterized using a custom PepSeq library with 7463 unique antigens, including 30 amino acid long peptides densely tiled across the entire EBOV-GP.

**Figure 2:**
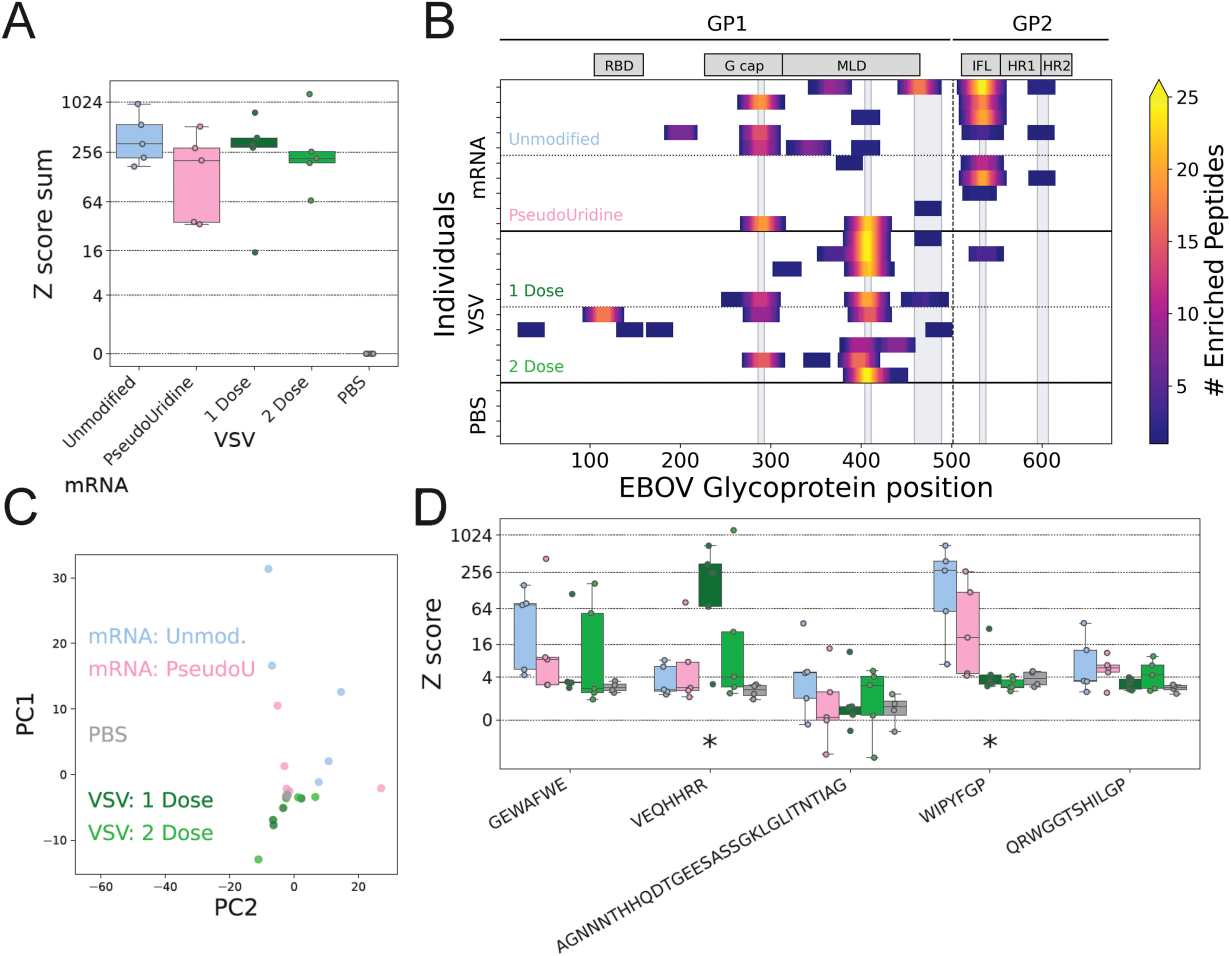
Linear epitope mapping of the polyclonal IgG responses to EBOV mRNA and rVSV vaccination. **(A)** Total anti-EBOV-GP IgG responses (sum of signal from all reactive peptides) measured using Pepseq and normalized to PBS control (for the serum described in **Figure 1**). All four vaccine conditions exhibited responses that were significantly different from the PBS controls (p<0.0013, Welch’s t-test), but no significant differences were observed between vaccine groups. **(B)** Distribution of enriched peptides (average Z score across replicates ≥10) across the EBOV-GP protein. 30mer peptides were used as assay probes. Numbers on the x-axis indicate animo acid position of the EBOV-GP, with a heat-map of numerical enrichment on the right. Vertical grey bars indicate the inferred core regions of the five most commonly reactive epitopes, which are also depicted in panel **(D)**. The black lines at the top of panel B indicate the two subunits of the EBOV-GP: GP1 and GP2. The grey boxes indicate the positions of a few functional domains: RBD = receptor binding domain, G cap = glycan cap, MLD = mucin-like domain, IFL = internal fusion loop, HR1, HR2 = heptad repeats 1 and 2. **(C)** Principal component (PC) analysis based on enrichment Z scores for 229 peptides, each exhibiting Z scores ≥10 in ≥3 individuals. Points are colored by vaccine group: “Unmod.” = Unmodified, “Modified” = N1MePsU modified. **(D)** Epitope-level maximum Z scores for five epitopes exhibiting strong reactivity (average Z score across replicates ≥10 in ≥3 individuals). Asterisks indicate epitopes at which we observed significant differences in reactivity between mRNA (n=10) and VSV (n=10) vaccinated animals (p=0.0277 for epitope VEQHHRR, p=0.00177 for epitope WIPYFGP; both by Welch’s t-test without corrections). Boxes are colored as in panel **(A)**.

An average of 656,295 (71,231 – 1,361,630) sequence reads were obtained per PepSeq assay, with two technical replicates per sample. Two replicates were excluded from further analysis due to low read counts (<30,000), but high-quality data was obtained for at least one replicate of each sample. For samples with two good replicates, a high level of concordance between replicates was observed (Pearson correlation coefficient ≥0.86, when considering peptides with Z score ≥ 10 in ≥1 replicates). Consistent with the ELISA results, significant anti-EBOV-GP IgG responses occurred in all vaccinated mice (compared to PBS controls), and the overall magnitude of these responses was not significantly different across vaccine platforms/formulations (**Figure 2A**). PepSeq detected IgG reactivity in at least one vaccinated individual against a total of 22 different epitopes across the EBOV GP (**Figure 2B**, **Supplementary Table S3**), with 3–12 significantly reactive epitopes per vaccinated individual (average = 7). This included epitopes within both GP1 and GP2, with most GP1 epitopes located within the glycan cap and mucin-like domain. Despite similarity in the overall magnitude of the antibody responses, peptide- and epitope-level analyses indicate that the mRNA and rVSV platforms biased antibody responses toward different epitopes (**Figures 2C, D**). In particular, there were significant differences in reactivity between mRNA and rVSV vaccinated mice at two epitopes (**Figure 2D**, Welch’s t-test). One epitope located in the mucin-like domain (“VEQHHRR”) exhibited significantly higher reactivity following rVSV vaccination (p=0.0277), while a second epitope in GP2 (“WIPYFGP”) exhibited significantly higher reactivity following mRNA vaccination (p=0.00177).

### 2.4 The EBOV mRNA vaccine protects type-I interferon deficient mice in a surrogate EBOV challenge model

**Figure 3:**
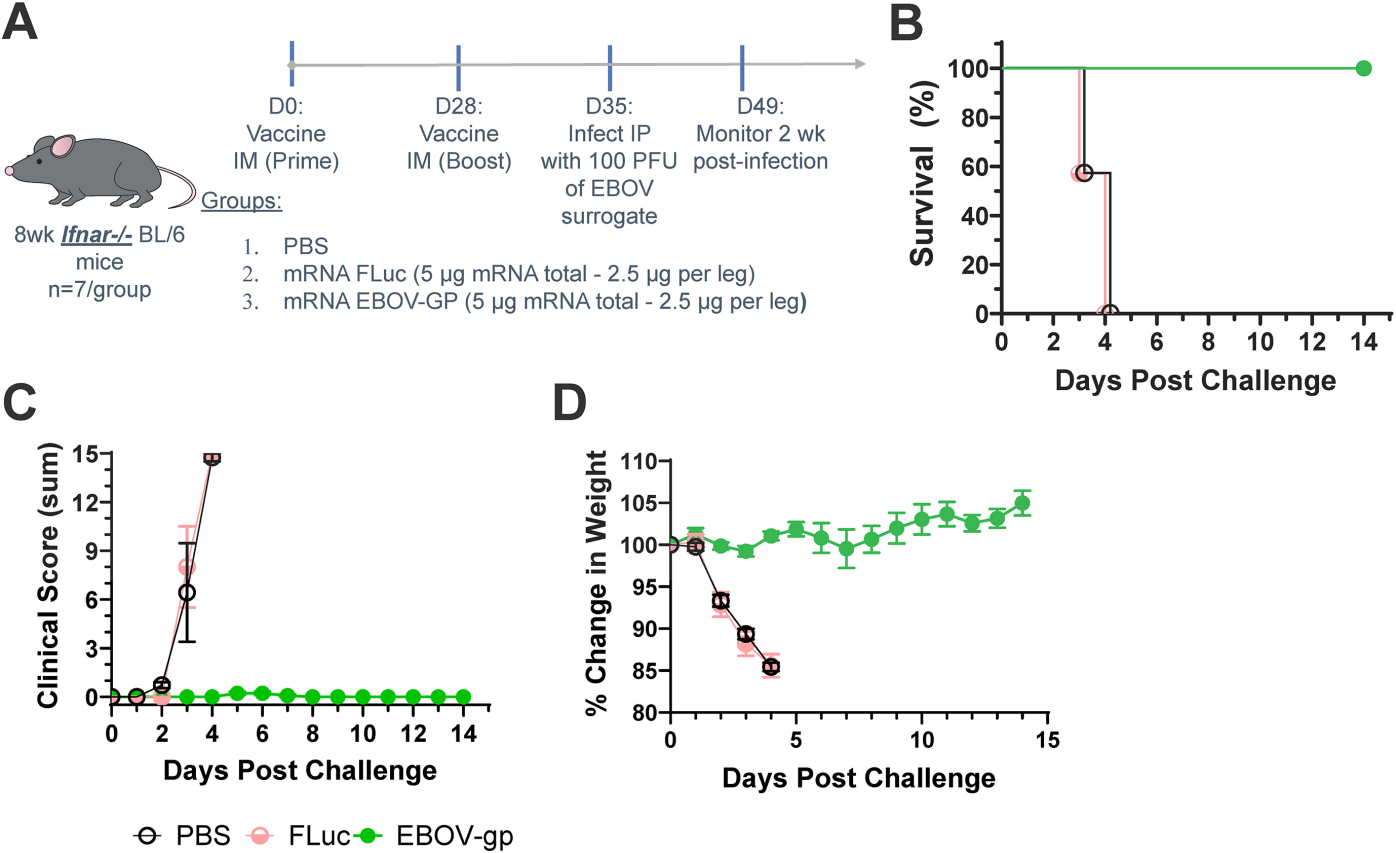
EBOV mRNA vaccine efficacy against surrogate virus challenge in a type I interferon knockout mouse model. **(A)** In this experimental design, *Ifnar*-/- mice on a C57BL/6J background were immunized intramuscularly with EBOV-GP mRNA, FLuc mRNA, or PBS. Mice were primed with the mRNA vaccines and boosted 28 days after prime. At 35 days post immunization, mice were intraperitoneally infected with a surrogate EBOV as a model of disease. Mice were monitored for 2 weeks after surrogate virus challenge. **(B–D)** Survival **(B)**, disease score **(C)**, and weight change **(D)** of immunized mice after disease challenge with surrogate EBOV.

*Ifnar* -/- mice lack type I interferon (IFN)-α/β receptors and are immunocompromised, particularly susceptible to viral infection, including wild type strains of EBOV and SUDV (69). In the event of a new outbreak strain of EBOV, this model could be valuable for rapidly assessing countermeasures in a cost-effective manner (70, 71). Additionally, the chimeric VSV expressing EBOV-GP which can induce EBOV-like disease in these immunocompromised mice and is thus used as a surrogate disease model (72, 73). Furthermore, surrogate EBOV (rVSV-EBOV-GP) is a risk group 2 agent that can be safely handled at BSL2, and is susceptible to neutralization by anti-EBOV GP antibodies, making it a viable EBOV model at ABSL2. The *Ifnar*-/- (C57BL/6 background) mouse model with surrogate EBOV challenge was used to interrogate protective humoral immunity generated by vaccination with EBOV-GP mRNA. Since the previous experiment in **Figure 1** showed similar immune responses to both unmodified and N1MePsU containing mRNA formulated into LNPs, unmodified mRNA was used in subsequent studies. Following a prime-boost regimen, *Ifnar* -/- mice were fully protected from lethal surrogate EBOV challenge with no clinical signs of infection or evident weight loss (**Figure 3**). As this model does not re-capitulate EBOV replication and pathogenicity is closer to what is observed following VSV infection, this provided evidence that the mRNA vaccine was able to effect protection through humoral responses to EBOV GP. Conversely, mice vaccinated with irrelevant FLuc mRNA or a mock condition displayed progressive clinical signs of illness and weight loss following challenge and universally succumbed to infection at day 3 or 4 post-virus administration.

### 2.5 mRNA and DNA EBOV vaccines protect mice from EBOD-like disease caused by pathogenic mouse-adapted EBOV challenge

**Figure 4:**
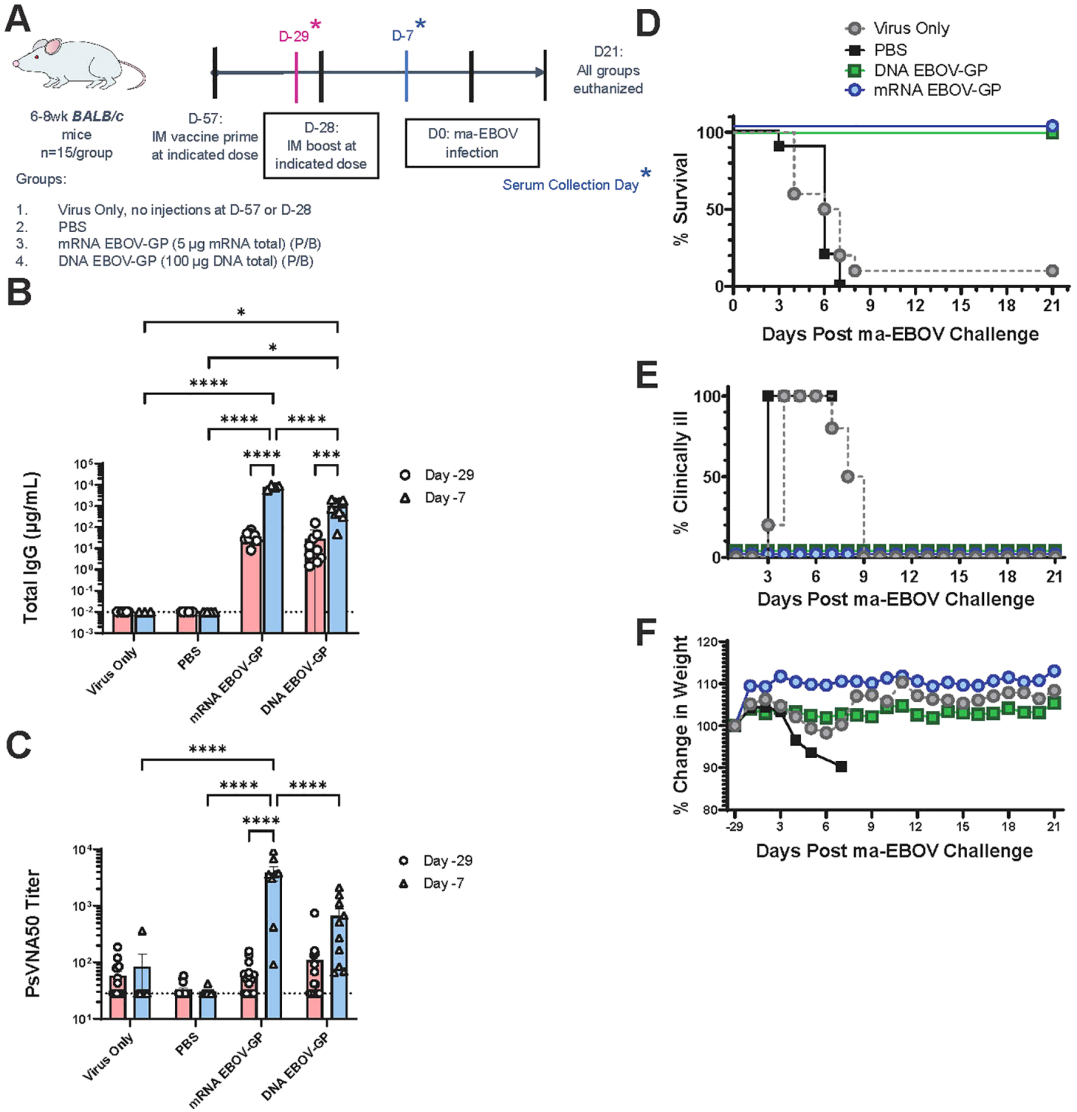
Protective efficacy of EBOV mRNA and DNA vaccines against mouse-adapted EBOV infection in wildtype mice. **(A)** In this experimental design, WT BALB/c mice were immunized with unmodified EBOV-GP mRNA, EBOV-GP DNA, or PBS via a prime+boost method with boost at 28 days post-prime. A group of BALB/c mice challenged with maEBOV but not provided any immunization injections was included as an additional control. A needle-free injection device was used to administer the DNA vaccine. Mice were bled one day before boost and one week before maEBOV challenge, so 27 days after prime and 21 days after boost, respectively. Sera was collected from blood. One month after boost, mice were challenged with maEBOV infection. All mice were monitored until euthanasia at 21 days post maEBOV disease challenge. **(B)** IgG ELISA for experimental mouse sera. Data are graphed by immunization group, with pink bars and circles indicating prime-induced immunity and blue bars and triangles indicating boost-induced immunity. **(C)** Neutralizing antibody titer from sera of the immunized mice. This pseudovirion neutralization assay (PsVNA) was quantified using a luciferase reporter assay, where decreased signal indicates increased neutralization by antibodies. **(D–F)** Survival **(D)**, clinical illness frequency **(E)**, and weight change **(F)** of mice after maEBOV challenge. Data is grouped as in **(B)** Bar graph dotted lines indicate limit of detection. Data are plotted in **(D–F)** as averages for all mice in the group. N = 15 per group. *p<0.05, ***p<0.001, ****p<0.0001 via 2-way ANOVA with Bonferroni’s correction for both B&C. Lack of illustrated comparison bar between comparison groups in **(B, C)***indicates no significance* between groups, where p>0.05. We omit non-significance comparison labels for clarity of figures.

BALB/c mice were immunized with either the EBOV mRNA LNP or an EBOV-GP DNA vaccine with a primary dose two months prior to challenge and a subsequent dose one month prior to challenge (**Figure 4A**). As the rVSV-EBOV-GP vaccine has been well characterized in BALB/c mice (15, 59–62) ethical constraints and limited maximum containment resources precluded inclusion in the ma-EBOV (mouse-adapted EBOV strain) challenge experiments. This is a limitation of this study that could potentially be addressed in the future work to allow more direct comparisons of vaccine platforms. However, the BALB/c model was utilized to allow cross-referencing of these nucleic acid vaccines with existing literature. The DNA was delivered using needle-free injection. One month following the booster vaccination, mice were challenged ma-EBOV. Prior to the booster and three weeks following the booster, serum was collected and measured for anti-EBOV titer and neutralizing capability. Both vaccines produced significantly increased antibody responses compared to PBS controls at both timepoints, and both vaccines produced significantly elevated titer following the booster dose (**Figure 4B**). However, EBOV neutralizing antibodies were only detected after the EBOV mRNA LNP or EBOV-GP DNA vaccine booster doses (**Figure 4C**). On average, the EBOV-GP specific IgG titers were 8.1-fold greater for the mRNA vaccine compared to the DNA vaccine, while the mRNA vaccine produced 5.8-fold greater neutralizing antibody titers than the DNA vaccine. Both the EBOV GP mRNA LNP and EBOV DNA vaccines were fully protective against lethal EBOD-like disease within our vaccinated mouse cohorts (n=15/group), showing no clinical signs of illness or weight loss through the 21 days post ma-EBOV challenge timepoint (**Figures 4D–F**). Conversely, mock vaccinated and unvaccinated controls had progressive weight loss, signs of illness starting at 3 days post-infection, and most met euthanasia criteria between 3- and 8-days post-infection. These two models of disease illustrate not only the capacity of our mRNA LNP platform to protect against EBOV and EBOV-like disease, but also the improved level of humoral-mediated protection.

## 3 Discussion

The rVSV, mRNA and DNA EBOV vaccines all generated robust humoral responses against EBOV-GP, demonstrating effective neutralization (**Figures 1B, C**, **4E, F**). However, vaccination with mRNA-EBOV-GP induced higher total and neutralizing antibody titers than rVSV-EBOV-GP, while achieving improved neutralizing titers compared to plasmid-based vaccines (**Figures 1B, C**, **4E, F**). There are subtle differences between EBOV strains, and different strains were the basis of the mRNA vaccine (Mayinga), the DNA vaccine (Kikwit), and the pseudovirus neutralization assay (Makona) presented here. While this is a factor to keep in mind during comparisons, the effects of these differences are likely limited. However, it is possible the difference in PsVNA50 titer observed between the mRNA and DNA vaccination seven days prior to challenge (**Figure 4E**) is attributable to strain variability. Notably, our epitope-resolved reactivity profiles (PepSeq) demonstrated that the mRNA and rVSV platforms bias antibody responses towards different epitopes within the EBOV-GP (**Figures 2C, D**). One of the epitopes preferentially targeted by mRNA vaccines (WIPYFGP) (**Figure 2D**) is part of an epitope known to induce both CD4 and CD8 T cell responses in mice after alphavirus replicon-EBOV-GP vaccination (74). This epitope is found in the internal fusion loop (IFL) of GP2, which is a target for many neutralizing monoclonal antibodies (75). Importantly, the residues in the IFL are highly conserved between Ebolavirus species (76, 77), indicating that our mRNA-EBOV-GP vaccine could target more than one Ebolavirus, though future studies will need to determine cross protection capacity.

The prime-boost strategy for rVSV-EBOV-GP vaccination produced greater splenic T cell-mediated responses compared to a single dose of rVSV-EBOV-GP or the prime+boost mRNA vaccines (**Figure 1D**). While ERBEVO is clinically administered as a single dose, completed and on-going clinical trials are assessing the benefits of rVSV-EBOV-GP booster doses (56, 57). In our hands, the single dose rVSV-EBOV-GP immunization (prime only) is used as an ERBEVO-like clinically relevant control for the memory timepoint, but up and coming studies could alter iterations of immunization timing and query further out memory timepoints when compared to an mRNA platform than is used here. The diminished EBOV-specific T cell responses against whole protein following EBOV mRNA vaccination (**Figure 1D**) is an interesting outcome considering other mRNA vaccines—against several viruses, including EBOV—have been able to generate apt T cell responses (78–80). A limitation to the cellular immunity data is the whole protein antigen approach utilized here. Alternatively, an EBOV-GP peptide library used for T cell stimulation can detect epitope specific responses to GP without any MHC processing and loading bias (37, 81). However, information on cellular immune responses to EBOV mRNA vaccines is limited, thus these data highlight the need for further research. Future efforts for platform comparisons between EBOV mRNA and other vaccines could include more detailed functional and phenotypic T cell assays, such as flow cytometry detection of polyfunctional T cells from peptide pool stimulation, antigen-specific T cell detection by EBOV-specific flow cytometry MHC-peptide tetramers, quantification and frequency of antigen-experienced T cells, cell-killing assays, and others (82–86). Significant effort has been put into mapping EBOV T cell epitopes (87–89), and future work will examine vaccine platforms for induction of T cell responses similar to those induced following natural infection.

Another important component of protection includes the formation of long-term immunological memory. Other mRNA vaccines have been shown to produce immunological memory cell pools which are key to the longevity of the immune response (68, 90, 91). Specifically, studies have illustrated that SARS-CoV-2 mRNA vaccines have the capacity to generate not only neutralizing antibodies, but also memory B and T cells (CD8 and CD4) (90). Unlike antibodies themselves, which wane over time, the presence of the memory populations, especially CD4 T cell and B cell memory, ensure the host is primed with pathogen-specific responses ready to address future pathogen challenges. The persistence of long-lived plasma cells, which produce antibodies, is also key for virus-specific immunity (92). VSV-based vaccines also share a capacity to retain a pool of antigen specific T cells, with tetramer-specific T cells being maintained via boosting regimens, but many analyses have focused on general function assessments. Further characterization of VSV-based vaccines, especially compared with mRNA and other platforms, is warranted (93–95). While this work compared the levels of antibodies present one to two months after immunization, the presence of memory immune populations that subsequently propagate EBOV-specific immune responses remains to be investigated across other vaccine platforms. Future studies addressing further numeration and characterization of both cellular and humoral memory should be conducted, especially at much later memory timepoints relative to boost, to ascertain the longevity of these platforms. These future studies could also extend to further flow cytometry phenotyping of the cells involved in the humoral response, such as memory B cells, plasma cells, germinal center B cells, and T follicular helper cells (83, 90, 96). Additionally, including parameters such as viremia and tissue viral load from pathogen-challenged mouse models may allow more granular comparisons of the correlates of vaccine efficacy.

Different prime-boost regimens, adjuvants, and modifications to the LNP composition of mRNA vaccines can enhance total immunogenicity and protection by modulating the immune responses. In the EBOV mRNA LNPs, neither the lipids nor non-specific mRNA molecules themselves provide protection against surrogate EBOV challenge, as demonstrated by dosing the *Ifnar* -/- mice with the Fluc mRNA LNP control (**Figure 3**). However, LNPs on their own can generate cytokine production and dendritic cell activation (97, 98). These inflammatory responses can be aided by the inclusion of toll-like receptor (TLR) agonists to stimulate pattern recognition receptors (PRRs) from host cells (99). These lipid nanoparticle-induced responses can accentuate and enhance antibody production and associated T follicular helper responses of mRNA vaccines and even protein subunit vaccines (100). The immunomodulatory effect of LNPs could potentially be exploited to generate more robust and durable adaptive immunity. Moreover, vaccines that induce primarily humoral responses may be important for some groups of immunocompromised patients. In immunocompromised patients of one study, two doses of the Moderna’s SARS-CoV-2 mRNA vaccine produced higher antibody titers and neutralization capacity than an adenovirus-vector vaccine (101). While a meta-analysis of the literature suggested similar findings, verification of this phenomena would require a higher number of adenovirus vaccine group candidates (67, 101). Furthermore, the usage of some replication competent live attenuated vaccines, including rVSV-EBOV-GP, is contraindicated for immunocompromised patients, as illustrated by lethal infection of this virus in *Ifnar-/-* mice (**Figure 3B**). Still, the immunocompromised nature of the *Ifnar* -/- mice along with the VSV based replication (and likely pathology) limits the usefulness of appreciating host responses to vaccines. For this reason, the mRNA vaccine was also tested in a ma-EBOV challenge model.

Lethal outbreaks of EBOD continue to occur (102). EBOV and related VHFs can affect both endemic populations as well as travelers to endemic regions. While an rVSV-based vaccine has been developed and approved for prophylactic use against EBOV, further understanding of advantages and disadvantages of multiple vaccine strategies are likely needed to ensure global health security against filoviruses. Here, we have provided a basis for the direct comparison of rVSV-vectored and nucleic acid EBOV vaccines. Expanding this work to include additional viral strains and in-depth assessment of correlates of protection will inform which vaccine platforms are best suited for specific pathogens, supporting the development of the next generation of vaccines with enhanced immune responses. While mRNA, DNA, and rVSV-vector vaccinations all offer varying degrees of protection, subtleties in their individual responses exist. Notably, the mRNA vaccine has demonstrated promising results in eliciting protective humoral responses. Indeed, the tested EBOV mRNA vaccine, which includes a modified nucleotide version used in commercial mRNA vaccines, demonstrated enhanced neutralization capacity through antibody responses and targeted unique regions of the EBOV-GP. These protective outcomes mirror what has been demonstrated recently with SARS-CoV-2 mRNA vaccines. For example, in mice, a codon-optimized mRNA LNP vaccine against the spike receptor binding domain of SARS-CoV-2 produced neutralizing antibodies, formed germinal centers where B cell maturation occurs, and generated an increase in memory B cells and class-switched B cells compared to a spike protein + adjuvant vaccine formulation (103). Parallel levels of humoral immunity from B and T follicular helper cells have also been seen in human SARS-CoV-2 cohorts and in murine studies against other pathogens like HIV and Zika (96, 104–106). Our observations of enhanced humoral immunity from the mRNA vaccine, when directly compared to a viral vector vaccine, are also supported by current literature focused on chikungunya virus vaccine development (82). These results support further investigation of mRNA vaccines for a role in the vaccine component of a comprehensive EBOD control plan. If nucleic acid vaccines were to be advanced to vaccine candidacy, future studies likely need to expand upon the groundwork laid here to allow better direct comparison to currently approved vaccines in EBOV challenge models including the more clinically relevant NHP models.

The DNA vaccine used in this study differed from earlier EBOV DNA vaccine studies in that the mode of delivery was needle-free injection. It is notable that the DNA vaccine was not LNP-formulated, and no adjuvant was included. We used needle-free injection because we, and others, have demonstrated that needle-free injection can enhance DNA vaccine potency and is more pragmatic than other technologies, such as particle-medicated epidermal delivery (gene gun) or electroporation (107, 108). Like the mRNA vaccine, protection was achieved after a prime and single boost. However, both total EBOV-GP specific IgG production (**Figure 4B**) and the neutralizing antibody response elicited by the mRNA vaccine (**Figure 4C**) were significantly higher than those induced by the DNA vaccine.

In summary, this work demonstrates that an mRNA vaccine platform against EBOV generates humoral immunity in mice that is comparable to or exceeds that of the currently licensed rVSV-EBOV-GP LAV. Additionally, this humoral immunity exhibited protective efficacy, which was confirmed against challenges from surrogate and mouse-adapted EBOV infections in mouse models.

## 4 Materials and methods

### 4.1 Vaccine preparation

#### 4.1.1 mRNA LNP vaccine production

The sequence of the GP from the EBOV strain Mayinga (Genbank: AY142960) was synthesized and cloned into a pT7 plasmid (VectorBuilder). The mRNA construct was synthesized using the HiScribe T7 mRNA kit with CleanCap Reagent AG (New England Biolabs) from a linearized plasmid template. The mRNA construct included an initiator methionine, a Kozak consensus sequence, and 5’ and 3’ untranslated regions (UTRs) as described previously with minor modifications (54). The human Igκ signal sequence replaced the EBOV GP signal sequence as described previously (6). As a negative control, a mRNA construct expressing the irrelevant antigen FLuc was created. mRNA was analyzed under denaturing conditions by gel electrophoresis to confirm size, quantified using a Qubit 3.0 fluorometer, and frozen at -70 °C until encapsulation. A NanoAssemblr Ignite nanoparticle formulation system (Cytiva; Marlborough, Mass.) was used to encapsulate the mRNA (500µg). Briefly, lipid components were prepared in ethanol at the following lipid molar ratio percentages: SM-102 (Cayman Chemical Ann Arbor, MI), 50%; DSPC, 10%; cholesterol, 38.5%; DMG-PEG2000, 1.5% (Avanti Research, Alabaster, AL) (54). mRNA cargo was prepared on ice in 50 mM citrate buffer, pH 3.5, until ready for formulation. Once both the organic and aqueous phases were ready, LNPs were formulated on the Ignite system at 12 mL/min total flow rate and a 3:1 aqueous to organic volume ratio. Post-formulation LNPs were dialyzed overnight against PBS at 4 °C. LNP samples were characterized with the Quant-it™ RiboGreen RNA Assay Kit (Invitrogen, Carlsbad, CA) to quantify mRNA content and assess encapsulation efficiency. Dynamic light scattering with the Malvern Zetasizer Nano ZS (Westborough, MA) was used to assess size and monodispersity. LNP mRNA vaccine aliquots were formulated with 8% sucrose, filter-sterilized, aliquoted, and stored at -80°C.

A549 cells were seeded, rested for 24 h, and transfected with 2 µg of mRNA using Lipofectamine MessengerMAX (Invitrogen, Waltham, MA) or TransIT (Mirus Bio, Madison, WI) transfection reagent according to the manufacturers’ direction. Following transfection, cells were incubated for one day. For immunofluorescence detection of EBOV GP expression, cells were fixed with 3.7% formaldehyde, washed with PBS, permeabilized with 0.1% Triton X-100, washed twice with PBS, blocked with SuperBlock blocking buffer (Thermo Scientific), stained with polyclonal rabbit anti-EBOV-GP at 1:500 dilution (Invitrogen, #PA5-117417), washed three times with PBS, stained with goat-anti-rabbit IgG secondary antibody, FITC at 1:1,000 dilution (Invitrogen, #A16118), washed three times with PBS and imaged using the GFP cube in a Cytation5 multi-mode reader (Biotek).

#### 4.1.2 rVSV-EBOV-GP vaccine production

The recombinant VSV expressing the EBOV-GP gene was derived from a full-length cDNA clone of VSV Indiana serotype 1, in which the VSV-G envelope protein has been replaced with EBOV-GP (rVSV-EBOV-GP). The EBOV-GP gene (GenBank: L11365) was cloned from a plasmid available from BEI Resources (Manassas, VA; catalog number NR-19814). The reverse genetics method used to rescue rVSV-EBOV-GP virus in 293T cells is described in **Supplementary Figure S2**.

An aliquot of rVSV-EBOV-GP was lysed in RIPA buffer with HALT protease inhibitor (Thermo Scientific, Waltman, MA) and total protein was quantified by BCA (Thermo Scientific, Waltman, MA). 45ng of protein was electrophoresed on an SDS-PAGE gel. Western blots were performed for EBOV-GP using a primary antibody (IBT Bioservices, Cat# 0301-015) at concentrations of 1:3,000. A goat anti-rabbit HRP secondary antibody (Cell Signaling Technology Cat #: 7074S) was used at a concentration of 1:10,000. Detection was performed via chemiluminescence on an Azure 600 imaging system (Dublin, CA).

#### 4.1.3 DNA vaccine production

To generate the EBOV-GP DNA vaccine, cDNA encoding the GP of EBOV-Kikwit 1995 (noted as EBOV-95) was encoded and cloned into a pWRG7077 plasmid by Aldevron (Fargo, ND). Vaccines were synthesized by Geneart (109, 110).

### 4.2 Cell lines

A549 (CCL-185), 293T (CRL-3216), and Vero CCL81 cell lines were obtained from ATCC (Manassas, VA). All cell lines were maintained at 37°C, 5% CO_2_. A549 cells were cultured in Dulbecco’s modified eagle medium (DMEM) with 4.5 g/L of glucose, 4 mM of L-glutamine, 110 of mg/L sodium pyruvate, 10% fetal bovine serum, 100 U/mL of penicillin, and 0.1 mg/mL of streptomycin. Vero cell lines were cultured in alpha-modified eagle medium (α-MEM) with 10% fetal bovine serum, and 1% penicillin/streptomycin.

### 4.3 *In vivo* experiments

#### 4.3.1 Immunogenicity studies

Six-week-old female C57BL/6 mice were obtained from Envigo and allocated to 5 groups with 5 mice per group at the start of the study (day 0). The groups received (1) mock vaccination with PBS, (2) 5 µg of LNPs containing EBOV-GP expressing mRNA with unmodified nucleotides, (3) 5 µg of LNPs containing EBOV-GP expressing mRNA substituting uridine with N1-methylpseudouridine (modified), or (4 & 5) 2.0×10^4^ PFUs rVSV-EBOV-GP. The dosing for rVSV-EBOV-GP was based on previously published literature (58, 71). This was the same for EBOV GP mRNA dosing, where dosing was based on previous mRNA immunization literature published on other pathogens (54, 111). To restrain mice for vaccinations, animals were anesthetized using inhaled anesthesia (i.e. isoflurane) using a VetEquip IMAC system set at 2-5%. All vaccinations were administered intramuscularly (IM) as a split dose of 50 µL per leg injected into the quadricep. 28-days after the first dose, groups 1, 2, 3, and 5 received an identical booster dose. One week later (35-days following the first dose), mice were euthanized in accordance with current American Veterinary Medical Association Guidelines for the Euthanasia of Animals 2020 and institute standard operating procedures. Endpoint blood collection was achieved via cardiac puncture. All animal work was conducted in accordance with protocols approved by the Lawrence Livermore National Laboratory (LLNL) Institution Animal Care and Use Committee.

#### 4.3.2 mRNA vaccination efficacy studies in Ifnar^-/-^ mice using surrogate EBOV challenge

Eight-week-old female Type-I interferon-α/β receptor deficient B6(Cg)-*Ifnar1^tm1.2Ees/J^* mice (*Ifnar* -/-) were obtained from the Jackson Laboratory (Bar Harbor, ME) and were vaccinated with 5 µg of LNPs containing EBOV-GP expressing mRNA(unmodified), split into two injections and administered IM into the quadricep. To restrain mice for vaccinations, animals were anesthetized using inhaled anesthesia (i.e. isoflurane) using a VetEquip IMAC system set at 2-5%. Two control groups were included, a group vaccinated with 5 µg of LNPs containing irrelevant FLuc expressing mRNA and a PBS mock vaccinated group. One month later, an identical booster vaccine was given. Seven days after the booster dose, mice were challenged with 100 PFU of surrogate EBOV (rVSV-EBOV-GP) administered by intraperitoneal (IP) injection as described previously (72). After viral challenge, the mice were followed for an additional two weeks for survival, changes in weight, and clinical score based on a 0–15 total point system with increased scoring indicating increased severity of signs and symptoms. Subjects that survived to end-of-study were euthanized 14 days post challenge. Euthanasia was performed under deep anesthesia by barbiturate (i.e., Euthasol Euthanasia Solution) overdose in accordance with current American Veterinary Medical Association Guidelines for the Euthanasia of Animals 2020 and institute standard operating procedures. All animal work was conducted in accordance with protocols approved by the Lawrence Livermore National Laboratory Institution Animal Care and Use Committee.

#### 4.3.3 mRNA LNP and DNA vaccine protection studies with authentic EBOV challenge

Six-to-8 week-old female BALB/c mice obtained from Charles River (Wilmington, MA) were vaccinated with the below regimens. For mRNA EBOV-GP treated mice, 5 µg of LNPs containing EBOV GP expressing mRNA (unmodified) intramuscularly. For EBOV-GP DNA immunized mice, DNA plasmid pWRG/EBOV Z76 (opt) (USARMIID) was formulated in PBS at a final stock concentration of 2 mg/mL EBOV DNA. For jet injection of mice, an adjustable PharmaJet Tropis device set at a 50 µL volume was used to deliver the DNA vaccine to the caudal thigh muscle. Due to the downscaling of this technology from human to mouse use, a higher dose of 100 µg DNA per mouse was used to improve immunogenicity. The mice were anesthetized for this procedure. To restrain mice for vaccinations, animals were anesthetized using inhaled anesthesia (i.e. isoflurane) using a VetEquip IMAC system set at 2-5%. The fur over the caudal thigh was shaved. The leg and thigh were held to support the leg when the disposable syringe was pressed against the muscle and discharged. A 50 µL liquid jet instantaneously penetrates the skin into the muscle. The needle-free injection device was a prototype variable-dose needle-free injection device (PharmaJet, Golden, CO). The prototype is a modified Tropis device capable of delivering volumes ranging from 20 to 100 µl for injection into small rodents. An additional group was similarly mock vaccinated with PBS. One month following the primary vaccination, an identical booster dose was administered. The day prior to administering the booster dose, and three weeks following the booster dose serum was collected by lateral saphenous vein of unanesthetized mice. Serum was collected for all animals on days -57 and -29 pre-challenge. Five animals per group were euthanized on day -21 pre-challenge, and spleen and serum were collected. Serum was collected for the remaining 10 animals per group on day -7 pre-challenge. Serum was collected and frozen at -80°C.

One month after the booster dose, mice were challenged via intraperitoneal administration with 100 PFU of mouse-adapted EBOV (ma-EBOV) IP (112). Following challenge, mice were assessed daily with the observation frequency increased to up to twice daily when clinical signs of illness were evident. Group mean body weights were obtained once daily during the first observation of the day. Disease scoring was quantified as percent of mice per group that showed signs of clinical illness. Mice that were unresponsive when provoked met criteria to be considered moribund and were euthanized. Animals that survived to until 21 days post-challenge, the end of the planned study, were euthanized. Euthanasia was performed under deep anesthesia by barbiturate (i.e., Euthasol Euthanasia Solution) overdose in accordance with current American Veterinary Medical Association Guidelines for the Euthanasia of Animals 2020 and institute standard operating procedures.

### 4.4 Immune response characterization

#### 4.4.1 Neutralization assay using a replication competent rVSV-EBOV-GP expressing GFP

Samples from **Figure 1C** were assayed as follows for neutralization capacity. Recombinant VSV-EBOV-GP expressing GFP (rVSV-EBOV-GFP) was derived from the cDNA clone of rVSV-EBOV-GP, where the VSV-P gene contained an N-terminal fusion to GFP. Vero CCL81 cells in supplemented alpha-MEM were seeded into black 96 well clear bottom plates (Corning) one day prior to incubation with virus and antibody. On the day of the assay, media was aspirated prior to addition of 0.5 PFU of VSV-EBOV-GFP, plus serially diluted serum from mice collected one week after boost immunization. Plates were left to incubate for 24 h before being fixed with 4% PFA in PBS followed by staining with DAPI nuclear stain at 1:1,000 dilution. Plates were then washed with PBS and remained in PBS while being read for percent fluorescence via Tecan Cyto Spark (Zurich, Switzerland). Titers are calculated from the reciprocal of the interpolated dilution that results in a 50% decrease in normalized percent infection.

#### 4.4.2 IFNγ T cell ELISpot assay

EBOV-specific T cell responses were measured with a murine IFN-γ enzymatic ELISpot ImmunoSpot kit (Cellular Technology Limited, Cleveland, OH) according to the manufacturer’s instructions. Briefly, a 96-well membrane plate was coated with capture antibody at 4°C overnight. The next day, plates were incubated with a TexMACS medium containing 10 µg EBOV-GP per well. Mouse spleens were collected, placed in RPMI + 10% FCS and 1% Pen/Strep, and processed into single cell suspensions. Prepared plates of EBOV-GP in TexMACS were plated with 250,000 splenocytes per well at a final volume of 200 µL/well for 48 h at 37°C, 5% CO_2_. On the final day of culture, plates were removed from incubation and washed prior to incubation with anti-IFNγ secondary antibody solution containing biotin for 2 h. Following this was an incubation of streptavidin-HRP tertiary solution for 30 minutes. Plates were developed by incubating with Reagent Blue and quenching with DDH_2_O. Plates were dried for imaging and quantification on a CTL ImmunoSpot analyzer (Cleveland, OH).

#### 4.4.3 Highly multiplexed serology with PepSeq

To quantify the magnitude and breadth of the antibody response generated by the vaccine platforms, we collected serum from the C57BL/6 mice at 1 week post boost immunization, or 35 days after the initial priming. Serum was assayed using a custom PepSeq library containing 30 amino acid long peptides tiled across the EBOV-GP protein using a method established previously (50, 51). The PepSeq platform for highly multiplexed serology facilitates simultaneous, quantitative measurement of antibody binding against 1000s of peptide antigens (50). Here, we used PepSeq to characterize IgG reactivity profiles across the full EBOV-GP with epitope-level resolution. Our PepSeq library included probes for each of the 677 unique, 30 amino acid peptides contained within the EBOV-GP immunogens used in this study. Additionally, because the presence of cysteines within PepSeq probes has been associated with reductions in signal (Elko et al., unpublished), for each wildtype peptide that included ≥1 cysteine, we also included a version of the peptide with all cysteines replaced with serines (222 additional peptides). DNA oligonucleotides encoding our peptides of interest were synthesized by Agilent Technologies, and these oligonucleotides were used as the starting material to synthesize PepSeq DNA-barcoded probes following our standard protocol (50).

PepSeq antibody binding assays were conducted as previously described (50). Briefly, each assay involved overnight incubation of 0.5 ml of mouse serum with 0.1 pmol of our PepSeq probes. Serum IgG was then precipitated using Streptococcal protein G bound to magnetic beads (Dynabeads, Invitrogen), non-binding PepSeq probes were washed away, and the relative abundance of each probe was quantified using PCR and high-throughput sequencing of the DNA portions of the PepSeq probes. Following PCR, a standard bead cleanup was performed, and products were individually quantified (Quant-It, Thermo Fisher), pooled, re-quantified (KAPA Library Quantification Kit, Roche) and sequenced on an Illumina NextSeq 1000 instrument (single end, 129 bp reads). Each serum sample was assayed, in duplicate, within a single 96-well plate, and to assess the expected relative abundance of each peptide, in the absence of antibody binding, we also included two buffer-only (“no serum”) negative control wells. Replicates with <30,000 mapped reads were excluded from further analysis resulting in the removal of two replicates, although at least one technical replicate of each sample was included in the analysis.

Sequencing data was processed using PepSIRF v1.7.0 (113), as well as associated Qiime2 plugins (114) and custom python scripts (https://github.com/LadnerLab/PepSIRF/tree/master/extensions). First, the reads were demultiplexed and assigned to peptides using the PepSIRF *demux* module, allowing for one mismatch in each index sequence and three mismatches in the variable DNA tag region. The PepSIRF *norm* module was then used to normalize counts to reads per million (RPM). RPM normalized read counts from two buffer-only controls were subsequently used to create bins for Z score calculation using the PepSIRF *bin* module. To normalize for different starting peptide abundances within each bin, reads were further normalized by subtracting the average RPM from the buffer-only controls (–diff option in *norm* module). Z scores were calculated using the PepSIRF *zscore* module using the 95% highest density interval within each bin.

Due to the dense tiling of our 30 amino acid peptides across the EBOV-GP (adjacent peptides overlap by 29 amino acids), we expect that many antibodies will bind to multiple, overlapping peptides contained in our PepSeq assay (i.e., many epitopes will be represented by multiple assay probes). Therefore, to more accurately measure the overall magnitude of the IgG response to vaccination, we first used our peptide-level reactivity measurements to define a set of antibody epitopes. Each reactive peptide (Z score ≥ 10) was assigned to an epitope, with overlapping peptides assigned to the same epitope. The core epitope was defined as the sequence shared across overlapping enriched peptides, and the core epitopes varied in length depending on the number and position of overlapping enriched peptides. To minimize lumping of overlapping epitopes, we set a minimum core epitope size of 7, and some peptides, at the junction of neighboring epitopes, were excluded from epitope-level analyses. For each sample, we used the maximum Z score, across the peptides that cover a given epitope, as a proxy for epitope-level reactivity. To generate a summary statistic of overall antibody reactivity against the EBOV-GP (“Z score sum”), we then summed these maximum Z scores across all epitopes at which a serum sample’s score differed significantly (more than two standard deviations above the mean) from the same scores observed across the mock-vaccinated animals (“PBS”). We used a custom python script (https://github.com/LadnerLab/PepSIRF/blob/master/extensions/epitopeLevelScores.py) for generating the epitope-level Z scores and Z score sum statistics.

To assess the overall similarity of PepSeq reactivity profiles across serum samples, we conducted a peptide level principal component analysis (PCA) using the scikit-learn python module (https://scikit-learn.org/stable/). This PCA was based on the average Z scores (across sample replicates) for 229 peptides against which we observed antibody reactivity (Z score ≥ 10) in ≥3 serum samples, regardless of the vaccine group to which the reactive samples belonged.

#### 4.4.4 Total IgG antibody quantification by ELISA

ELISA plates were coated with 100 µl of EBOV-GP (2µg/ml in PBS) in each well, sealed, and incubated at 4°C overnight. Plates were washed with washing buffer (PBS with 0.01% Tween 20) 3 times, then blocked with 150 µL/well Blocking Buffer (10X PBS with 10% BSA and 0.5% Tween-20) for 2 hours at room temperature. After decanting, wells were washed 3 times with washing buffer. C57BL/6 or BALB/c mouse serum samples were diluted 1:40 in blocking buffer. Samples were plated in duplicate (50 µL per well), and incubated overnight at 4°C. Wells were washed 3 times with washing buffer and 50 µL/well of anti-mouse IgG-HRP (1:3,000) and incubated for 1 hour at room temperature. Plates were washed 4 times and 50 µL/well of TMB substrate was added with the plates incubating at room temperature for 15 minutes in the dark. The reaction was stopped with 50 µL 1M HCl, and absorbance was measured at 450 nm. Samples were normalized to a concentration-defined dilution series of an anti-EBOV GP monoclonal antibody (clone 13F6) to generate values of antibody as concentrations of µg/mL.

#### 4.4.5 Pseudovirus neutralization assay

Serum specimens from the experiments performed in **Figure 4C** were evaluated for the presence of neutralizing antibodies using a pseudovirion neutralization assay (PsVNA) (115–118). The PsVNA utilizes engineered VSV that expresses a luciferase reporter gene in the place of the virus G envelope glycoprotein genes. Ebola pseudovirions (PsVs) were produced using: pWRG/EBOV-76 (Makona). The neutralization assay was performed by combining 4000 focus forming units with serum (1:40–3,125,000 dilution range) in the presence of guinea pig complement (5%; Cedarlane, Burlington, NC, USA) and incubated overnight at 2–8°C. This mixture was then added to ATCC Vero-76 (CRL-1587, Manassas, VA) cell monolayers in clear bottom black-walled 96-well microtiter plates. The plates were incubated 18–24 h and then media was removed, lysis luciferase reagent (Promega, Madison, WI, USA) was added and flash luminescence data was acquired using a luminometer (Tecan M200 Pro microplate reader). If sera contain antibodies that prevent the PsV from attaching to and/or entering cells, then the reporter activity is neutralized.

### 4.5 Statistical analysis

Statistical analyses illustrated in bar graphs were run using GraphPad Prism version 10.4.0. Statistics were run as indicated in figure legends. Neutralization titers are interpolated from 4-parameter curves using GraphPad Prism 10 (GraphPad, San Diego, CA, USA). The reciprocal of the interpolated dilution that results in a 50% decrease in luciferase activity is the PsVNA50. This was performed in duplicate with a geometric mean being taken of the two values obtained. For figures where one and two-way non-parametric ANOVA statistical comparisons are shown, lack of illustrated comparison bar between comparison groups indicates *no significance* between groups, where p>0.05. We omit these lines and “ns” labels for clarity of figures. For the comparison of EBOV glycoprotein epitopes between VSV and mRNA vaccines, 5 independent Welch’s t-tests were performed, one for each epitope, without correction for false discovery rate to prioritize minimization of type II errors.

## Data Availability

The raw data supporting the conclusions of this article will be made available by the authors, without undue reservation.

## Author disclaimer

Opinions, interpretations, conclusions, and recommendations are those of the authors and are not necessarily endorsed by the U.S. Army. Research was conducted under an IACUC approved protocol in compliance with the Animal Welfare Act, PHS Policy, and other Federal statutes and regulations relating to animals and experiments involving animals. The facility where this research was conducted is accredited by the Association for Assessment and Accreditation of Laboratory Animal Care, International and adheres to principles stated in the Guide for the Care and Use of Laboratory Animals, National Research Council, 2011.
