# Supplementary material for: An mRNA vaccine encoding the Ebola virus glycoprotein induces high neutralizing antibody titers and provides strong protection against lethal infections in mouse models
Source: Front Immunol. 2026 Jan 5;16:1682418. doi: 10.3389/fimmu.2025.1682418 (PMC12813019; doi:10.3389/fimmu.2025.1682418)
Supplement: Supplementary file 1 [file DataSheet1.docx]

Supplementary Material

##
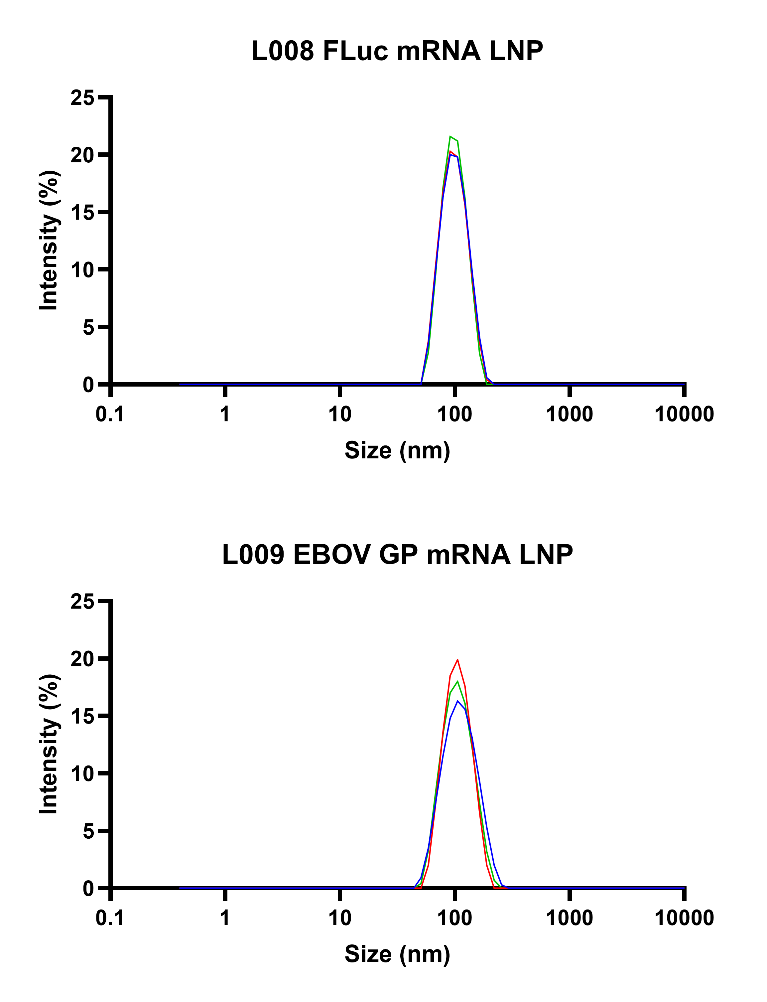

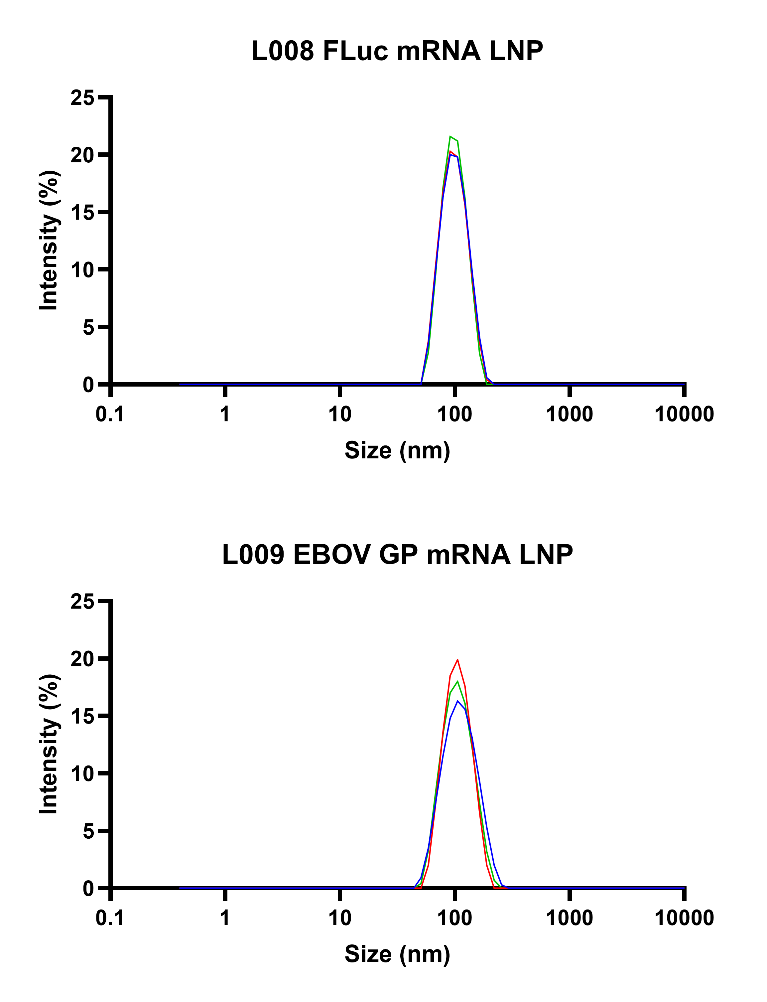

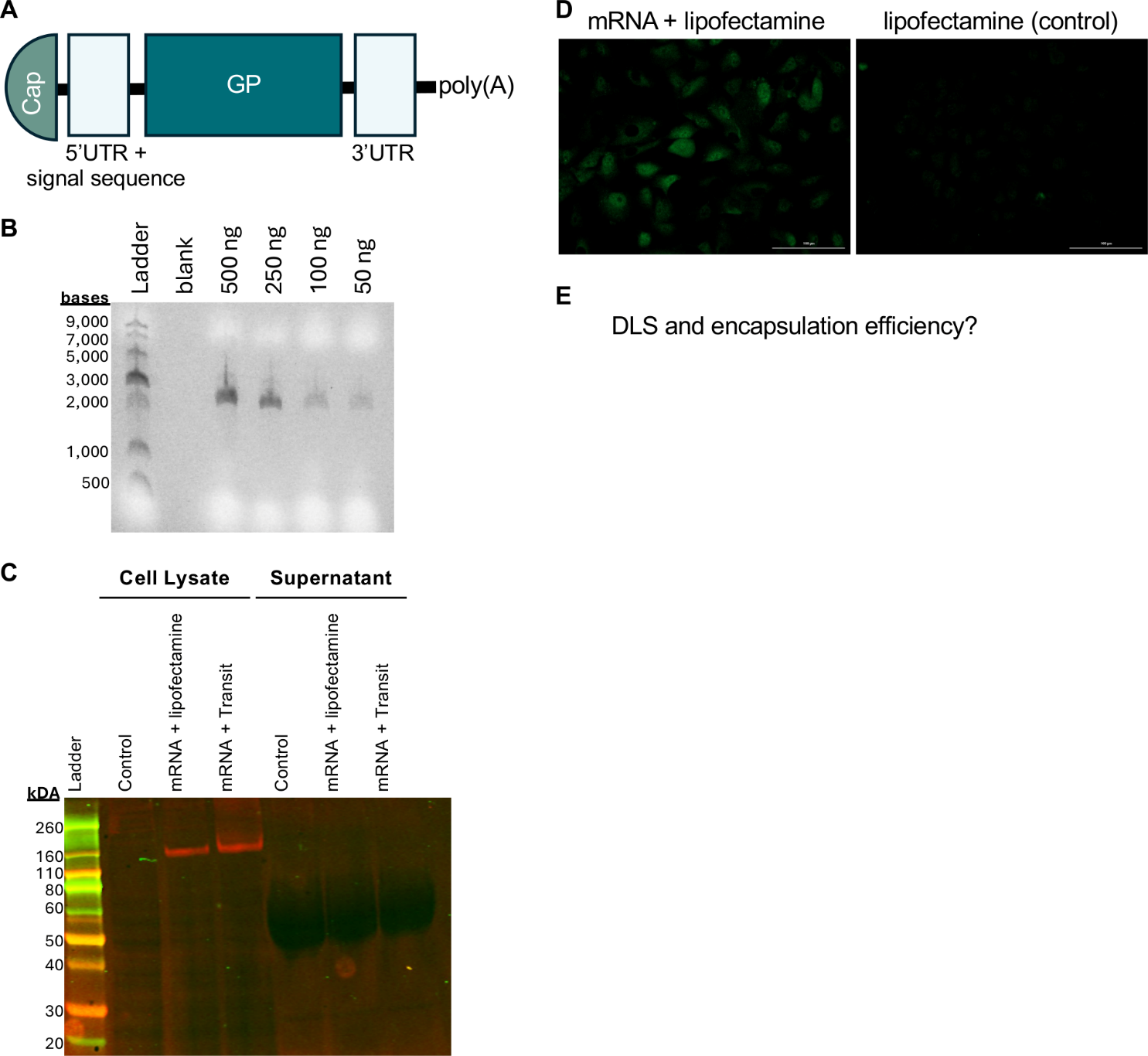

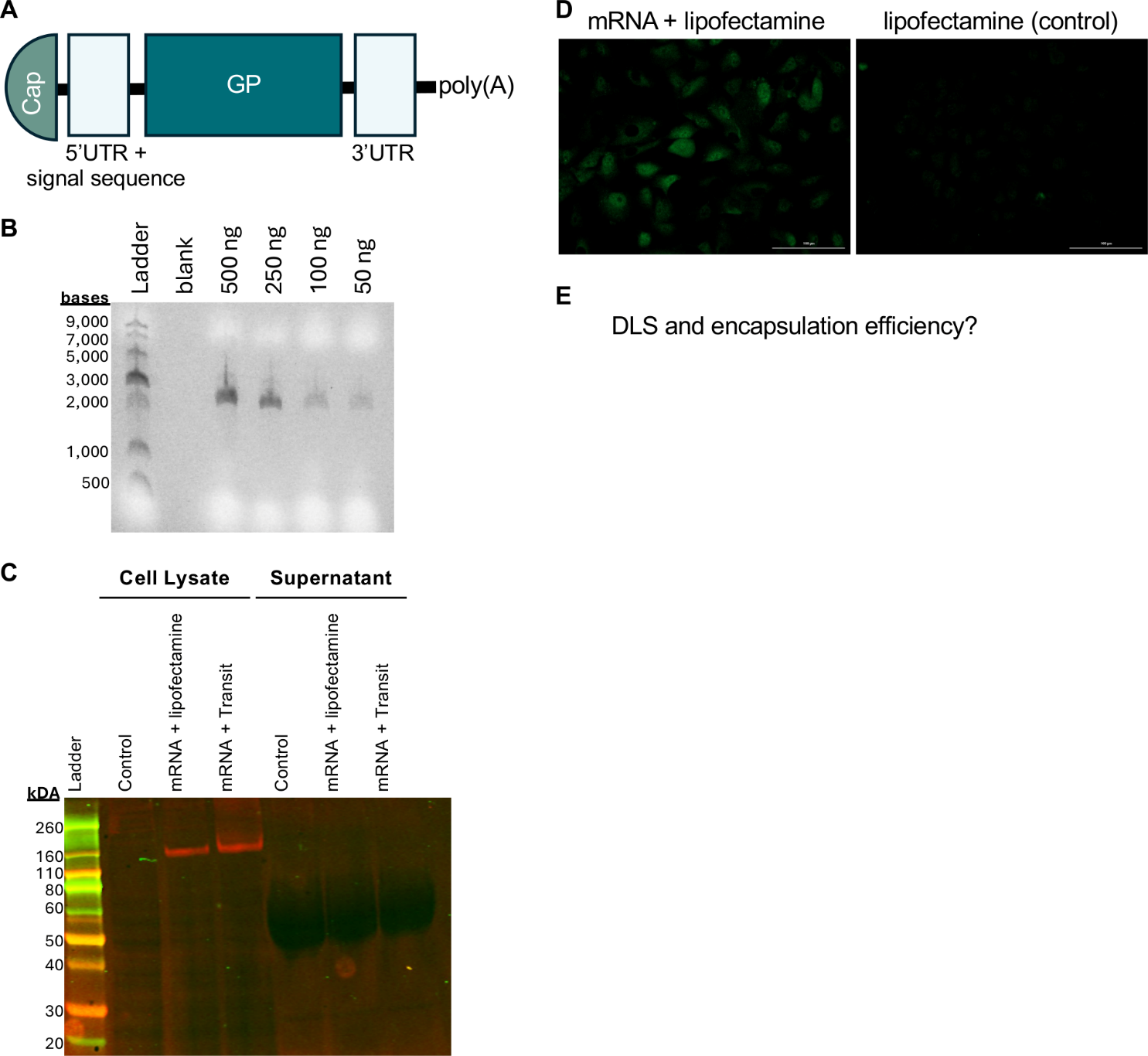
Supplementary Figures

**Figure S1.** Design, encapsulation, and *in vitro* expression of the mRNA vaccine. (**A**) Schematic of the mRNA candidate encoding Ebola virus glycoprotein (GP). (**B**) The concentration of mRNA constructs was determined using a Qubit fluorometer and were used to detect the RNA product using agarose gel electrophoresis run under denaturing conditions and stained with Syber Green. The expected size of the mRNA is 2,152 bases (not including the poly-A tail adding about 100 additional bases). (**C**) A549 cells were transfected with mRNA using either lipofectamine (lanes 3, 6) or transIT (lanes 4, 7) transfection reagent 24 hours prior to collection. Untreated A549 controls were included (lanes 2, 4). A fluorescent ladder was loaded in lane 1. Cell lysates (lanes 2, 3, 4) and supernatants (lanes5, 6, 7) were analyzed by western blot analysis with polyclonal anti-EBOV-GP antibody. (**D**) Protein production was verified by immunofluorescent microscopy of A549 cells transfected with mRNA 24 hours prior to fixing and staining with poly-clonal anti-EBOV antibody (left) or cells transfected with lipofectamine without mRNA (control, right). Twenty-four hours later, cells were labeled with an antibody against GP and then with an FITC-conjugated secondary antibody. Scale bar is 100 µm. (**E**) Dynamic light scattering was conducted to assess the size and monodispersity of EBOV-GP mRNA LNPs (top) and control FLuc containing mRNA LNPs (bottom). Measurements were done in triplicate.

**A**

**
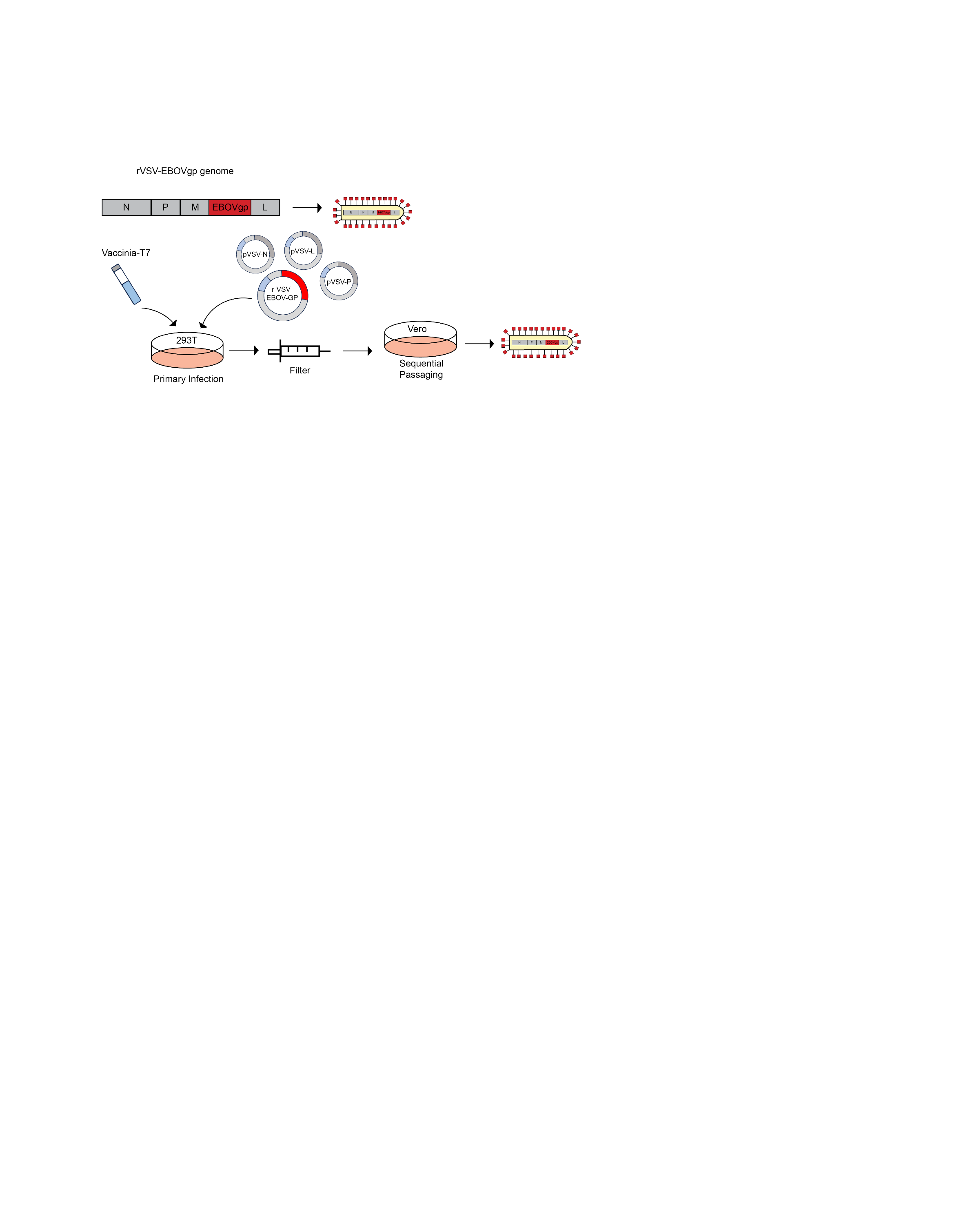
**

**B**

**Figure S2.** Validation of EBOV-GP antigen expression in the rVSV platform. **(A)** A schematic depicting the reverse genetics system used to rescue rVSV-EBOV-GP virus in 293T cells and expanded in Vero-CCL81 cells. **(B)** EBOV-GP expression measured by western blot analysis from rVSV-EBOV-GP infected cell lysates. rVSV-EBOV-GP was expanded over Vero-CCL81 cells and purified by ultracentrifugation at 30,000 RPM in an SW32Ti rotor for 1 hour and 15 minutes at 4*°*C. Purified virus was resuspended in a minimal volume of PBS, lysed in RIPA buffer with HALT protease inhibitor, and total protein was quantified by BCA. Samples were diluted to 4.5ng/µL and 10µL of each sample was loaded on an SDS-PAGE gel. Western blots were performed for EBOV-GP. A VSV-VEEV construct was included as non-EBOV-GP purified VSV control. Primary antibody was used at concentrations of 1:3000 for EBOV-GP in 5% milk-TBST. Secondary antibody was used at a concentration of 1:10000 in 5% milk-TBST. Detection was performed via chemiluminescence.


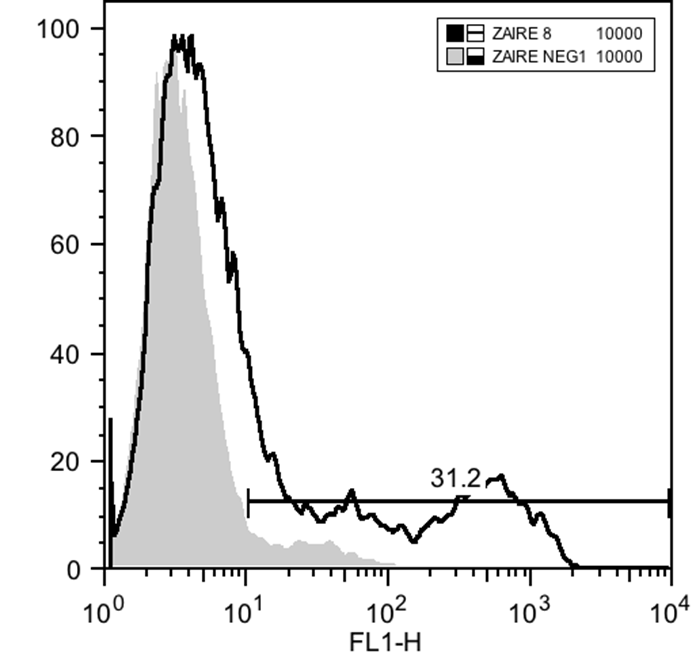


**Figure S3.** Validation of EBOV-GP antigen expression in the DNA platform. 15µg of EBOV-GP expressing DNA was transfected into HEK 293T cells using Fugene 6. The next day, cells were stained with anti-Ebola 76 rabbit sera (USAMRIID), followed by an anti-rabbit 488 secondary (Invitrogen). Untransfected control cells are shown as gray-shaded area. Based on cut-off, 31.2% of cells transfected with the EBOV-95 DNA were expressing protein bound by the anti-Ebola rabbit sera.

**
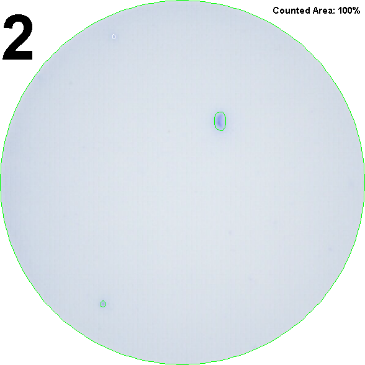
**

**
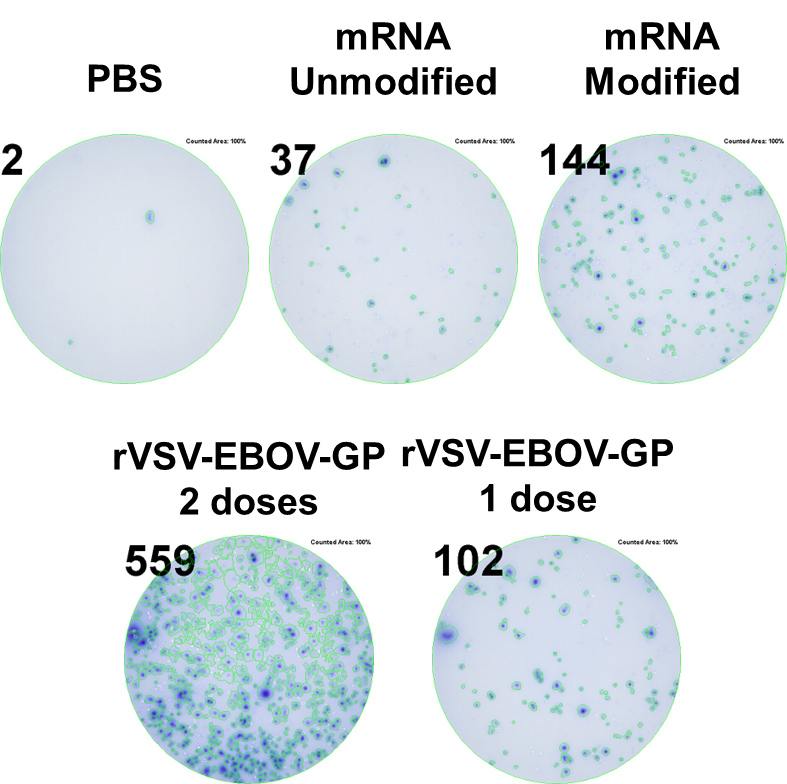
**

**Figure S4.** Representative well images from 48hr EBOV-GP stimulation of immunized splenocytes in Figure 1D. C57BL/6 mouse spleens from mice immunized in Figure 1 were plated at 2105 cells/well with EBOV-GP in L-glutamine supplemented CTL media for 48 hours at 37C/5% CO2 incubation conditions. Plates were developed with CTL mouse IFN single color enzymatic assay per manufacturer’s instructions. Plates were dried prior to imaging and counting using a CTL Immunospot analyzer. Numbers on the top right corner are Spot Forming Unit (SFU) counts calculated by the Immunospot analyzer. Green outlines around purple/blue spots are software-generated outlines of regions included in the count.

**Table S1**. DNA sequences used in *in vitro* transcription vector

| **Element** | **Sequence** |
| --- | --- |
| 5’UTR | GGGAAATAAGAGAGAAAAGAAGAGTAAGAAGAAATATAAGA |
| Kozak | GCCGCCACC |
| Start | ATG |
| Igκ | GAGACCCCCGCCCAGCTGCTGTTCCTGCTGCTGCTGTGGCTGCCCGACACCACCGGC |
| Ebola virus glycoprotein | ATCCCCCTGGGCGTGATCCACAACTCCACCCTGCAGGTGTCCGACGTGGACAAGCTGGTGTGCCGCGACAAGCTGTCCTCCACCAACCAGCTGCGCTCCGTGGGCCTGAACCTGGAGGGCAACGGCGTGGCCACCGACGTGCCCTCCGCCACCAAGCGCTGGGGCTTCCGCTCCGGCGTGCCCCCCAAGGTGGTGAACTACGAGGCCGGCGAGTGGGCCGAGAACTGCTACAACCTGGAGATCAAGAAGCCCGACGGCTCCGAGTGCCTGCCCGCCGCCCCCGACGGCATCCGCGGCTTCCCCCGCTGCCGCTACGTGCACAAGGTGTCCGGCACCGGCCCCTGCGCCGGCGACTTCGCCTTCCACAAGGAGGGCGCCTTCTTCCTGTACGACCGCCTGGCCTCCACCGTGATCTACCGCGGCACCACCTTCGCCGAGGGCGTGGTGGCCTTCCTGATCCTGCCCCAGGCCAAGAAGGACTTCTTCTCCTCCCACCCCCTGCGCGAGCCCGTGAACGCCACCGAGGACCCCTCCTCCGGCTACTACTCCACCACCATCCGCTACCAGGCCACCGGCTTCGGCACCAACGAGACCGAGTACCTGTTCGAGGTGGACAACCTGACCTACGTGCAGCTGGAGTCCCGCTTCACCCCCCAGTTCCTGCTGCAGCTGAACGAGACCATCTACACCTCCGGCAAGCGCTCCAACACCACCGGCAAGCTGATCTGGAAGGTGAACCCCGAGATCGACACCACCATCGGCGAGTGGGCCTTCTGGGAGACCAAGAAGAACCTGACCCGCAAGATCCGCTCCGAGGAGCTGTCCTTCACCGTGGTGTCCAACGGCGCCAAGAACATCTCCGGCCAGTCCCCCGCCCGCACCTCCTCCGACCCCGGCACCAACACCACCACCGAGGACCACAAGATCATGGCCTCCGAGAACTCCTCCGCCATGGTGCAGGTGCACTCCCAGGGCCGCGAGGCCGCCGTGTCCCACCTGACCACCCTGGCCACCATCTCCACCTCCCCCCAGTCCCTGACCACCAAGCCCGGCCCCGACAACTCCACCCACAACACCCCCGTGTACAAGCTGGACATCTCCGAGGCCACCCAGGTGGAGCAGCACCACCGCCGCACCGACAACGACTCCACCGCCTCCGACACCCCCTCCGCCACCACCGCCGCCGGCCCCCCCAAGGCCGAGAACACCAACACCTCCAAGTCCACCGACTTCCTGGACCCCGCCACCACCACCTCCCCCCAGAACCACTCCGAGACCGCCGGCAACAACAACACCCACCACCAGGACACCGGCGAGGAGTCCGCCTCCTCCGGCAAGCTGGGCCTGATCACCAACACCATCGCCGGCGTGGCCGGCCTGATCACCGGCGGCCGCCGCACCCGCCGCGAGGCCATCGTGAACGCCCAGCCCAAGTGCAACCCCAACCTGCACTACTGGACCACCCAGGACGAGGGCGCCGCCATCGGCCTGGCCTGGATCCCCTACTTCGGCCCCGCCGCCGAGGGCATCTACACCGAGGGCCTGATGCACAACCAGGACGGCCTGATCTGCGGCCTGCGCCAGCTGGCCAACGAGACCACCCAGGCCCTGCAGCTGTTCCTGCGCGCCACCACCGAGCTGCGCACCTTCTCCATCCTGAACCGCAAGGCCATCGACTTCCTGCTGCAGCGCTGGGGCGGCACCTGCCACATCCTGGGCCCCGACTGCTGCATCGAGCCCCACGACTGGACCAAGAACATCACCGACAAGATCGACCAGATCATCCACGACTTCGTGGACAAGACCCTGCCCGACCAGGGCGACAACGACAACTGGTGGACCGGCTGGCGCCAGTGGATCCCCGCCGGCATCGGCGTGACCGGCGTGATCATCGCCGTGATCGCCCTGTTCTGCATCTGCAAGTTCGTGTTC |
| Stop | TGATAATAG |
| 3’UTR | GCTGGAGCCTCGGTGGCCATGCTTCTTGCCCCTTGGGCCTCCCCCCAGCCCCTCCTCCCCTTCCTGCACCCGTACCCCCGTGGTCTTTGAATAAAGTCTGA |

**Table S2**. mRNA lipid nanoparticle encapsulation efficiency

| **Sample Description** | **Lot #** | **Encapsulated RNA (ug/mL)** | **Encapsulation Efficiency** | **Final LNP RNA (ug/mL)** | **Aliquot Amt (ug)** | **Z-Avg (nm)** | **PDI** |
| --- | --- | --- | --- | --- | --- | --- | --- |
| FLuc mRNA LNP | L008 | 380.80 | 98.13% | 120 | 12 | 95.16 | 0.051 |
| EBOV GP mRNA LNP | L009 | 361.24 | 98.33% | 120 | 12 | 101.73 | 0.084 |

**Table S3**. Ebola virus Glycoprotein epitopes exhibiting PepSeq reactivity following

vaccination. Each dotted line in the peptides column represents an individual peptide sequence.

| **Epitope** | **Peptides*** |
| --- | --- |
| FLWVIILFQRTFSIPLGVIHNSTLQVSDVD | FLWVIILFQRTFSIPLGVIHNSTLQVSDVD |
| CYNLEIKKPDGSEC | \| VPPKVVNYEAGEWAENCYNLEIKKPDGSEC \| \| --- \| \| PPKVVNYEAGEWAENCYNLEIKKPDGSECL \| \| PKVVNYEAGEWAENCYNLEIKKPDGSECLP \| \| KVVNYEAGEWAENCYNLEIKKPDGSECLPA \| \| VVNYEAGEWAENCYNLEIKKPDGSECLPAA \| \| VNYEAGEWAENCYNLEIKKPDGSECLPAAP \| \| NYEAGEWAENCYNLEIKKPDGSECLPAAPD \| \| YEAGEWAENCYNLEIKKPDGSECLPAAPDG \| \| EAGEWAENCYNLEIKKPDGSECLPAAPDGI \| \| AGEWAENCYNLEIKKPDGSECLPAAPDGIR \| \| GEWAENCYNLEIKKPDGSECLPAAPDGIRG \| \| EWAENCYNLEIKKPDGSECLPAAPDGIRGF \| \| WAENCYNLEIKKPDGSECLPAAPDGIRGFP \| \| AENCYNLEIKKPDGSECLPAAPDGIRGFPR \| \| ENCYNLEIKKPDGSECLPAAPDGIRGFPRC \| \| NCYNLEIKKPDGSECLPAAPDGIRGFPRCR \| \| CYNLEIKKPDGSECLPAAPDGIRGFPRCRY \| |
| YDRLASTVIYRGTTFAEGVVAFLILPQAKK | YDRLASTVIYRGTTFAEGVVAFLILPQAKK |
| FSSHPLREPVNATEDPSS | \| AFLILPQAKKDFFSSHPLREPVNATEDPSS \| \| --- \| \| FLILPQAKKDFFSSHPLREPVNATEDPSSG \| \| LILPQAKKDFFSSHPLREPVNATEDPSSGY \| \| ILPQAKKDFFSSHPLREPVNATEDPSSGYY \| \| LPQAKKDFFSSHPLREPVNATEDPSSGYYS \| \| PQAKKDFFSSHPLREPVNATEDPSSGYYST \| \| QAKKDFFSSHPLREPVNATEDPSSGYYSTT \| \| AKKDFFSSHPLREPVNATEDPSSGYYSTTI \| \| KKDFFSSHPLREPVNATEDPSSGYYSTTIR \| \| KDFFSSHPLREPVNATEDPSSGYYSTTIRY \| \| DFFSSHPLREPVNATEDPSSGYYSTTIRYQ \| \| FFSSHPLREPVNATEDPSSGYYSTTIRYQA \| \| FSSHPLREPVNATEDPSSGYYSTTIRYQAT \| |
| ESRFTPQFLLQLNETIYTSGKRSNTTGKLI | ESRFTPQFLLQLNETIYTSGKRSNTTGKLI |
| GEWAFWE | \| SGKRSNTTGKLIWKVNPEIDTTIGEWAFWE \| \| --- \| \| GKRSNTTGKLIWKVNPEIDTTIGEWAFWET \| \| KRSNTTGKLIWKVNPEIDTTIGEWAFWETK \| \| RSNTTGKLIWKVNPEIDTTIGEWAFWETKK \| \| SNTTGKLIWKVNPEIDTTIGEWAFWETKKN \| \| NTTGKLIWKVNPEIDTTIGEWAFWETKKNL \| \| TTGKLIWKVNPEIDTTIGEWAFWETKKNLT \| \| TGKLIWKVNPEIDTTIGEWAFWETKKNLTR \| \| GKLIWKVNPEIDTTIGEWAFWETKKNLTRK \| \| KLIWKVNPEIDTTIGEWAFWETKKNLTRKI \| \| LIWKVNPEIDTTIGEWAFWETKKNLTRKIR \| \| IWKVNPEIDTTIGEWAFWETKKNLTRKIRS \| \| WKVNPEIDTTIGEWAFWETKKNLTRKIRSE \| \| KVNPEIDTTIGEWAFWETKKNLTRKIRSEE \| \| VNPEIDTTIGEWAFWETKKNLTRKIRSEEL \| \| NPEIDTTIGEWAFWETKKNLTRKIRSEELS \| \| PEIDTTIGEWAFWETKKNLTRKIRSEELSF \| \| EIDTTIGEWAFWETKKNLTRKIRSEELSFT \| \| IDTTIGEWAFWETKKNLTRKIRSEELSFTV \| \| DTTIGEWAFWETKKNLTRKIRSEELSFTVV \| \| TTIGEWAFWETKKNLTRKIRSEELSFTVVS \| \| TIGEWAFWETKKNLTRKIRSEELSFTVVSN \| \| IGEWAFWETKKNLTRKIRSEELSFTVVSNG \| \| GEWAFWETKKNLTRKIRSEELSFTVVSNGA \| |
| EELSFTVVSNGAKNISGQSPARTSSDPGTN | EELSFTVVSNGAKNISGQSPARTSSDPGTN |
| HKIMASEN | \| NISGQSPARTSSDPGTNTTTEDHKIMASEN \| \| --- \| \| ISGQSPARTSSDPGTNTTTEDHKIMASENS \| \| SGQSPARTSSDPGTNTTTEDHKIMASENSS \| \| GQSPARTSSDPGTNTTTEDHKIMASENSSA \| \| QSPARTSSDPGTNTTTEDHKIMASENSSAM \| \| SPARTSSDPGTNTTTEDHKIMASENSSAMV \| \| PARTSSDPGTNTTTEDHKIMASENSSAMVQ \| \| ARTSSDPGTNTTTEDHKIMASENSSAMVQV \| \| RTSSDPGTNTTTEDHKIMASENSSAMVQVH \| \| TSSDPGTNTTTEDHKIMASENSSAMVQVHS \| \| SSDPGTNTTTEDHKIMASENSSAMVQVHSQ \| \| SDPGTNTTTEDHKIMASENSSAMVQVHSQG \| \| DPGTNTTTEDHKIMASENSSAMVQVHSQGR \| \| PGTNTTTEDHKIMASENSSAMVQVHSQGRE \| \| GTNTTTEDHKIMASENSSAMVQVHSQGREA \| \| TNTTTEDHKIMASENSSAMVQVHSQGREAA \| \| NTTTEDHKIMASENSSAMVQVHSQGREAAV \| \| TTTEDHKIMASENSSAMVQVHSQGREAAVS \| \| TTEDHKIMASENSSAMVQVHSQGREAAVSH \| \| TEDHKIMASENSSAMVQVHSQGREAAVSHL \| \| EDHKIMASENSSAMVQVHSQGREAAVSHLT \| \| DHKIMASENSSAMVQVHSQGREAAVSHLTT \| \| HKIMASENSSAMVQVHSQGREAAVSHLTTL \| |
| AAVSHLTTLATISTS | \| ENSSAMVQVHSQGREAAVSHLTTLATISTS \| \| --- \| \| NSSAMVQVHSQGREAAVSHLTTLATISTSP \| \| SSAMVQVHSQGREAAVSHLTTLATISTSPQ \| \| SAMVQVHSQGREAAVSHLTTLATISTSPQS \| \| AMVQVHSQGREAAVSHLTTLATISTSPQSL \| \| MVQVHSQGREAAVSHLTTLATISTSPQSLT \| \| VQVHSQGREAAVSHLTTLATISTSPQSLTT \| \| QVHSQGREAAVSHLTTLATISTSPQSLTTK \| \| VHSQGREAAVSHLTTLATISTSPQSLTTKP \| \| HSQGREAAVSHLTTLATISTSPQSLTTKPG \| \| SQGREAAVSHLTTLATISTSPQSLTTKPGP \| \| QGREAAVSHLTTLATISTSPQSLTTKPGPD \| \| GREAAVSHLTTLATISTSPQSLTTKPGPDN \| \| REAAVSHLTTLATISTSPQSLTTKPGPDNS \| \| EAAVSHLTTLATISTSPQSLTTKPGPDNST \| \| AAVSHLTTLATISTSPQSLTTKPGPDNSTH \| |
| STSPQSLTTKPGPDNSTHNTPVYKLDISEA | STSPQSLTTKPGPDNSTHNTPVYKLDISEA |
| TPVYKLDISEATQ | \| SPQSLTTKPGPDNSTHNTPVYKLDISEATQ \| \| --- \| \| PQSLTTKPGPDNSTHNTPVYKLDISEATQV \| \| QSLTTKPGPDNSTHNTPVYKLDISEATQVE \| \| SLTTKPGPDNSTHNTPVYKLDISEATQVEQ \| \| LTTKPGPDNSTHNTPVYKLDISEATQVEQH \| \| TTKPGPDNSTHNTPVYKLDISEATQVEQHH \| |
| VEQHHRR | \| PVYKLDISEATQVEQHHRRTDNDSTASDTP \| \| --- \| \| VYKLDISEATQVEQHHRRTDNDSTASDTPS \| \| YKLDISEATQVEQHHRRTDNDSTASDTPSA \| \| KLDISEATQVEQHHRRTDNDSTASDTPSAT \| \| LDISEATQVEQHHRRTDNDSTASDTPSATT \| \| DISEATQVEQHHRRTDNDSTASDTPSATTA \| \| ISEATQVEQHHRRTDNDSTASDTPSATTAA \| \| SEATQVEQHHRRTDNDSTASDTPSATTAAG \| \| EATQVEQHHRRTDNDSTASDTPSATTAAGP \| \| ATQVEQHHRRTDNDSTASDTPSATTAAGPP \| \| TQVEQHHRRTDNDSTASDTPSATTAAGPPK \| \| QVEQHHRRTDNDSTASDTPSATTAAGPPKA \| \| VEQHHRRTDNDSTASDTPSATTAAGPPKAE \| |
| HHRRTDNDSTASDTPSATTAAGPPKAENTN | HHRRTDNDSTASDTPSATTAAGPPKAENTN |
| GPPKAENTNTSKSTDFLDPA  GPPKAENTNTSKSTDFLDPA | \| SDTPSATTAAGPPKAENTNTSKSTDFLDPA \| \| --- \| \| DTPSATTAAGPPKAENTNTSKSTDFLDPAT \| \| TPSATTAAGPPKAENTNTSKSTDFLDPATT \| \| PSATTAAGPPKAENTNTSKSTDFLDPATTT \| \| SATTAAGPPKAENTNTSKSTDFLDPATTTS \| \| ATTAAGPPKAENTNTSKSTDFLDPATTTSP \| \| TTAAGPPKAENTNTSKSTDFLDPATTTSPQ \| \| TAAGPPKAENTNTSKSTDFLDPATTTSPQN \| \| AAGPPKAENTNTSKSTDFLDPATTTSPQNH \| \| AGPPKAENTNTSKSTDFLDPATTTSPQNHS \| \| GPPKAENTNTSKSTDFLDPATTTSPQNHSE \| |
| SETAGNNNTHHQDT | \| STDFLDPATTTSPQNHSETAGNNNTHHQDT \| \| --- \| \| TDFLDPATTTSPQNHSETAGNNNTHHQDTG \| \| DFLDPATTTSPQNHSETAGNNNTHHQDTGE \| \| FLDPATTTSPQNHSETAGNNNTHHQDTGEE \| \| LDPATTTSPQNHSETAGNNNTHHQDTGEES \| \| DPATTTSPQNHSETAGNNNTHHQDTGEESA \| \| PATTTSPQNHSETAGNNNTHHQDTGEESAS \| \| ATTTSPQNHSETAGNNNTHHQDTGEESASS \| \| TTTSPQNHSETAGNNNTHHQDTGEESASSG \| \| TTSPQNHSETAGNNNTHHQDTGEESASSGK \| \| TSPQNHSETAGNNNTHHQDTGEESASSGKL \| \| SPQNHSETAGNNNTHHQDTGEESASSGKLG \| \| PQNHSETAGNNNTHHQDTGEESASSGKLGL \| \| QNHSETAGNNNTHHQDTGEESASSGKLGLI \| \| NHSETAGNNNTHHQDTGEESASSGKLGLIT \| \| HSETAGNNNTHHQDTGEESASSGKLGLITN \| \| SETAGNNNTHHQDTGEESASSGKLGLITNT \| |
| AGNNNTHHQDTGEESASSGKLGLITNTIAG | AGNNNTHHQDTGEESASSGKLGLITNTIAG |
| EESASSGKLGLITNTIAGVAGLITG | \| HQDTGEESASSGKLGLITNTIAGVAGLITG \| \| --- \| \| QDTGEESASSGKLGLITNTIAGVAGLITGG \| \| DTGEESASSGKLGLITNTIAGVAGLITGGR \| \| TGEESASSGKLGLITNTIAGVAGLITGGRR \| \| GEESASSGKLGLITNTIAGVAGLITGGRRT \| \| EESASSGKLGLITNTIAGVAGLITGGRRTR \| |
| LITGGRRTRKEA  LITGGRRTRKEA  LITGGRRTRKEA | \| ASSGKLGLITNTIAGVAGLITGGRRTRKEA \| \| --- \| \| SSGKLGLITNTIAGVAGLITGGRRTRKEAI \| \| SGKLGLITNTIAGVAGLITGGRRTRKEAIV \| \| GKLGLITNTIAGVAGLITGGRRTRKEAIVN \| \| KLGLITNTIAGVAGLITGGRRTRKEAIVNA \| \| LGLITNTIAGVAGLITGGRRTRKEAIVNAQ \| \| GLITNTIAGVAGLITGGRRTRKEAIVNAQP \| \| LITNTIAGVAGLITGGRRTRKEAIVNAQPK \| \| ITNTIAGVAGLITGGRRTRKEAIVNAQPKC \| \| TNTIAGVAGLITGGRRTRKEAIVNAQPKCN \| \| NTIAGVAGLITGGRRTRKEAIVNAQPKCNP \| \| TIAGVAGLITGGRRTRKEAIVNAQPKCNPN \| \| IAGVAGLITGGRRTRKEAIVNAQPKCNPNL \| \| AGVAGLITGGRRTRKEAIVNAQPKCNPNLH \| \| GVAGLITGGRRTRKEAIVNAQPKCNPNLHY \| \| VAGLITGGRRTRKEAIVNAQPKCNPNLHYW \| \| AGLITGGRRTRKEAIVNAQPKCNPNLHYWT \| \| GLITGGRRTRKEAIVNAQPKCNPNLHYWTT \| \| LITGGRRTRKEAIVNAQPKCNPNLHYWTTQ \| \| ASSGKLGLITNTIAGVAGLITGGRRTRREA \| \| SSGKLGLITNTIAGVAGLITGGRRTRREAI \| \| SGKLGLITNTIAGVAGLITGGRRTRREAIV \| \| GKLGLITNTIAGVAGLITGGRRTRREAIVN \| \| KLGLITNTIAGVAGLITGGRRTRREAIVNA \| \| LGLITNTIAGVAGLITGGRRTRREAIVNAQ \| \| GLITNTIAGVAGLITGGRRTRREAIVNAQP \| \| LITNTIAGVAGLITGGRRTRREAIVNAQPK \| \| ITNTIAGVAGLITGGRRTRREAIVNAQPKC \| \| TNTIAGVAGLITGGRRTRREAIVNAQPKCN \| \| NTIAGVAGLITGGRRTRREAIVNAQPKCNP \| \| TIAGVAGLITGGRRTRREAIVNAQPKCNPN \| \| IAGVAGLITGGRRTRREAIVNAQPKCNPNL \| \| AGVAGLITGGRRTRREAIVNAQPKCNPNLH \| \| GVAGLITGGRRTRREAIVNAQPKCNPNLHY \| \| VAGLITGGRRTRREAIVNAQPKCNPNLHYW \| \| AGLITGGRRTRREAIVNAQPKCNPNLHYWT \| \| GLITGGRRTRREAIVNAQPKCNPNLHYWTT \| \| LITGGRRTRREAIVNAQPKCNPNLHYWTTQ \| \| ITNTIAGVAGLITGGRRTRKEAIVNAQPKS \| \| TNTIAGVAGLITGGRRTRKEAIVNAQPKSN \| \| NTIAGVAGLITGGRRTRKEAIVNAQPKSNP \| \| TIAGVAGLITGGRRTRKEAIVNAQPKSNPN \| \| IAGVAGLITGGRRTRKEAIVNAQPKSNPNL \| \| AGVAGLITGGRRTRKEAIVNAQPKSNPNLH \| \| GVAGLITGGRRTRKEAIVNAQPKSNPNLHY \| \| VAGLITGGRRTRKEAIVNAQPKSNPNLHYW \| \| AGLITGGRRTRKEAIVNAQPKSNPNLHYWT \| \| GLITGGRRTRKEAIVNAQPKSNPNLHYWTT \| \| LITGGRRTRKEAIVNAQPKSNPNLHYWTTQ \| \| ITNTIAGVAGLITGGRRTRREAIVNAQPKS \| \| TNTIAGVAGLITGGRRTRREAIVNAQPKSN \| \| NTIAGVAGLITGGRRTRREAIVNAQPKSNP \| \| TIAGVAGLITGGRRTRREAIVNAQPKSNPN \| \| IAGVAGLITGGRRTRREAIVNAQPKSNPNL \| \| AGVAGLITGGRRTRREAIVNAQPKSNPNLH \| \| GVAGLITGGRRTRREAIVNAQPKSNPNLHY \| \| VAGLITGGRRTRREAIVNAQPKSNPNLHYW \| \| AGLITGGRRTRREAIVNAQPKSNPNLHYWT \| \| GLITGGRRTRREAIVNAQPKSNPNLHYWTT \| \| LITGGRRTRREAIVNAQPKSNPNLHYWTTQ \| |
| NAQPKSNPNLHYWTTQDEGA | \| GRRTRKEAIVNAQPKCNPNLHYWTTQDEGA \| \| --- \| \| RRTRKEAIVNAQPKCNPNLHYWTTQDEGAA \| \| RTRKEAIVNAQPKCNPNLHYWTTQDEGAAI \| \| TRKEAIVNAQPKCNPNLHYWTTQDEGAAIG \| \| RKEAIVNAQPKCNPNLHYWTTQDEGAAIGL \| \| KEAIVNAQPKCNPNLHYWTTQDEGAAIGLA \| \| EAIVNAQPKCNPNLHYWTTQDEGAAIGLAW \| \| AIVNAQPKCNPNLHYWTTQDEGAAIGLAWI \| \| IVNAQPKCNPNLHYWTTQDEGAAIGLAWIP \| \| VNAQPKCNPNLHYWTTQDEGAAIGLAWIPY \| \| NAQPKCNPNLHYWTTQDEGAAIGLAWIPYF \| \| GRRTRREAIVNAQPKCNPNLHYWTTQDEGA \| \| RRTRREAIVNAQPKCNPNLHYWTTQDEGAA \| \| RTRREAIVNAQPKCNPNLHYWTTQDEGAAI \| \| TRREAIVNAQPKCNPNLHYWTTQDEGAAIG \| \| RREAIVNAQPKCNPNLHYWTTQDEGAAIGL \| \| REAIVNAQPKCNPNLHYWTTQDEGAAIGLA \| \| GRRTRKEAIVNAQPKSNPNLHYWTTQDEGA \| \| RRTRKEAIVNAQPKSNPNLHYWTTQDEGAA \| \| RTRKEAIVNAQPKSNPNLHYWTTQDEGAAI \| \| TRKEAIVNAQPKSNPNLHYWTTQDEGAAIG \| \| RKEAIVNAQPKSNPNLHYWTTQDEGAAIGL \| \| KEAIVNAQPKSNPNLHYWTTQDEGAAIGLA \| \| EAIVNAQPKSNPNLHYWTTQDEGAAIGLAW \| \| AIVNAQPKSNPNLHYWTTQDEGAAIGLAWI \| \| IVNAQPKSNPNLHYWTTQDEGAAIGLAWIP \| \| VNAQPKSNPNLHYWTTQDEGAAIGLAWIPY \| \| NAQPKSNPNLHYWTTQDEGAAIGLAWIPYF \| \| GRRTRREAIVNAQPKSNPNLHYWTTQDEGA \| \| RRTRREAIVNAQPKSNPNLHYWTTQDEGAA \| \| RTRREAIVNAQPKSNPNLHYWTTQDEGAAI \| \| TRREAIVNAQPKSNPNLHYWTTQDEGAAIG \| \| RREAIVNAQPKSNPNLHYWTTQDEGAAIGL \| \| REAIVNAQPKSNPNLHYWTTQDEGAAIGLA \| |
| WIPYFGP  WIPYFGP  WIPYFGP | \| QPKCNPNLHYWTTQDEGAAIGLAWIPYFGP \| \| --- \| \| PKCNPNLHYWTTQDEGAAIGLAWIPYFGPA \| \| KCNPNLHYWTTQDEGAAIGLAWIPYFGPAA \| \| CNPNLHYWTTQDEGAAIGLAWIPYFGPAAE \| \| NPNLHYWTTQDEGAAIGLAWIPYFGPAAEG \| \| PNLHYWTTQDEGAAIGLAWIPYFGPAAEGI \| \| NLHYWTTQDEGAAIGLAWIPYFGPAAEGIY \| \| LHYWTTQDEGAAIGLAWIPYFGPAAEGIYI \| \| HYWTTQDEGAAIGLAWIPYFGPAAEGIYIE \| \| YWTTQDEGAAIGLAWIPYFGPAAEGIYIEG \| \| WTTQDEGAAIGLAWIPYFGPAAEGIYIEGL \| \| TTQDEGAAIGLAWIPYFGPAAEGIYIEGLM \| \| TQDEGAAIGLAWIPYFGPAAEGIYIEGLMH \| \| QDEGAAIGLAWIPYFGPAAEGIYIEGLMHN \| \| DEGAAIGLAWIPYFGPAAEGIYIEGLMHNQ \| \| EGAAIGLAWIPYFGPAAEGIYIEGLMHNQD \| \| GAAIGLAWIPYFGPAAEGIYIEGLMHNQDG \| \| AAIGLAWIPYFGPAAEGIYIEGLMHNQDGL \| \| AIGLAWIPYFGPAAEGIYIEGLMHNQDGLI \| \| IGLAWIPYFGPAAEGIYIEGLMHNQDGLIC \| \| GLAWIPYFGPAAEGIYIEGLMHNQDGLICG \| \| LAWIPYFGPAAEGIYIEGLMHNQDGLICGL \| \| AWIPYFGPAAEGIYIEGLMHNQDGLICGLR \| \| WIPYFGPAAEGIYIEGLMHNQDGLICGLRQ \| \| LHYWTTQDEGAAIGLAWIPYFGPAAEGIYT \| \| HYWTTQDEGAAIGLAWIPYFGPAAEGIYTE \| \| YWTTQDEGAAIGLAWIPYFGPAAEGIYTEG \| \| WTTQDEGAAIGLAWIPYFGPAAEGIYTEGL \| \| TTQDEGAAIGLAWIPYFGPAAEGIYTEGLM \| \| TQDEGAAIGLAWIPYFGPAAEGIYTEGLMH \| \| QDEGAAIGLAWIPYFGPAAEGIYTEGLMHN \| \| DEGAAIGLAWIPYFGPAAEGIYTEGLMHNQ \| \| EGAAIGLAWIPYFGPAAEGIYTEGLMHNQD \| \| GAAIGLAWIPYFGPAAEGIYTEGLMHNQDG \| \| AAIGLAWIPYFGPAAEGIYTEGLMHNQDGL \| \| AIGLAWIPYFGPAAEGIYTEGLMHNQDGLI \| \| IGLAWIPYFGPAAEGIYTEGLMHNQDGLIC \| \| GLAWIPYFGPAAEGIYTEGLMHNQDGLICG \| \| LAWIPYFGPAAEGIYTEGLMHNQDGLICGL \| \| AWIPYFGPAAEGIYTEGLMHNQDGLICGLR \| \| WIPYFGPAAEGIYTEGLMHNQDGLICGLRQ \| \| QPKSNPNLHYWTTQDEGAAIGLAWIPYFGP \| \| PKSNPNLHYWTTQDEGAAIGLAWIPYFGPA \| \| KSNPNLHYWTTQDEGAAIGLAWIPYFGPAA \| \| SNPNLHYWTTQDEGAAIGLAWIPYFGPAAE \| \| IGLAWIPYFGPAAEGIYIEGLMHNQDGLIS \| \| GLAWIPYFGPAAEGIYIEGLMHNQDGLISG \| \| LAWIPYFGPAAEGIYIEGLMHNQDGLISGL \| \| AWIPYFGPAAEGIYIEGLMHNQDGLISGLR \| \| WIPYFGPAAEGIYIEGLMHNQDGLISGLRQ \| \| IGLAWIPYFGPAAEGIYTEGLMHNQDGLIS \| \| GLAWIPYFGPAAEGIYTEGLMHNQDGLISG \| \| LAWIPYFGPAAEGIYTEGLMHNQDGLISGL \| \| AWIPYFGPAAEGIYTEGLMHNQDGLISGLR \| \| WIPYFGPAAEGIYTEGLMHNQDGLISGLRQ \| |
| QRWGGTSHILGP  QRWGGTSHILGP | \| TELRTFSILNRKAIDFLLQRWGGTCHILGP \| \| --- \| \| ELRTFSILNRKAIDFLLQRWGGTCHILGPD \| \| LRTFSILNRKAIDFLLQRWGGTCHILGPDC \| \| RTFSILNRKAIDFLLQRWGGTCHILGPDCC \| \| TFSILNRKAIDFLLQRWGGTCHILGPDCCI \| \| FSILNRKAIDFLLQRWGGTCHILGPDCCIE \| \| SILNRKAIDFLLQRWGGTCHILGPDCCIEP \| \| ILNRKAIDFLLQRWGGTCHILGPDCCIEPH \| \| LNRKAIDFLLQRWGGTCHILGPDCCIEPHD \| \| NRKAIDFLLQRWGGTCHILGPDCCIEPHDW \| \| RKAIDFLLQRWGGTCHILGPDCCIEPHDWT \| \| KAIDFLLQRWGGTCHILGPDCCIEPHDWTK \| \| AIDFLLQRWGGTCHILGPDCCIEPHDWTKN \| \| IDFLLQRWGGTCHILGPDCCIEPHDWTKNI \| \| DFLLQRWGGTCHILGPDCCIEPHDWTKNIT \| \| FLLQRWGGTCHILGPDCCIEPHDWTKNITD \| \| LLQRWGGTCHILGPDCCIEPHDWTKNITDK \| \| LQRWGGTCHILGPDCCIEPHDWTKNITDKI \| \| QRWGGTCHILGPDCCIEPHDWTKNITDKID \| \| TELRTFSILNRKAIDFLLQRWGGTSHILGP \| \| ELRTFSILNRKAIDFLLQRWGGTSHILGPD \| \| LRTFSILNRKAIDFLLQRWGGTSHILGPDS \| \| RTFSILNRKAIDFLLQRWGGTSHILGPDSS \| \| TFSILNRKAIDFLLQRWGGTSHILGPDSSI \| \| FSILNRKAIDFLLQRWGGTSHILGPDSSIE \| \| SILNRKAIDFLLQRWGGTSHILGPDSSIEP \| \| ILNRKAIDFLLQRWGGTSHILGPDSSIEPH \| \| LNRKAIDFLLQRWGGTSHILGPDSSIEPHD \| \| NRKAIDFLLQRWGGTSHILGPDSSIEPHDW \| \| RKAIDFLLQRWGGTSHILGPDSSIEPHDWT \| \| KAIDFLLQRWGGTSHILGPDSSIEPHDWTK \| \| AIDFLLQRWGGTSHILGPDSSIEPHDWTKN \| \| IDFLLQRWGGTSHILGPDSSIEPHDWTKNI \| \| DFLLQRWGGTSHILGPDSSIEPHDWTKNIT \| \| FLLQRWGGTSHILGPDSSIEPHDWTKNITD \| \| LLQRWGGTSHILGPDSSIEPHDWTKNITDK \| \| LQRWGGTSHILGPDSSIEPHDWTKNITDKI \| \| QRWGGTSHILGPDSSIEPHDWTKNITDKID \| |
| IRGFPRCRYVHKVSGTGPCAGDFAFHKEGA | IRGFPRSRYVHKVSGTGPSAGDFAFHKEGA |

* PepSeq library peptides considered, for each epitope, when calculating epitope-level reactivity.
